# The Potential of Hair Matrix for Biomarker Analysis in Schizophrenia

**DOI:** 10.3390/ijms26178718

**Published:** 2025-09-07

**Authors:** Enric Rubio-Contreras, Nora Guasch-Capella, Albert Martínez-Pinteño, David Olivares-Berjaga, Constanza Morén

**Affiliations:** 1 Department of Basic and Clinical Practice, University of Barcelona, 08036 Barcelona, Spain; erubioc01@gmail.com (E.R.-C.); olivares99@ub.edu (D.O.-B.); 2 Barcelona Clínic Schizophrenia Unit (BCSU), Department of Psychiatry, Institute of Neuroscience, Hospital Clínic of Barcelona, University of Barcelona, 08036 Barcelona, Spain; noguasch@recerca.clinic.cat; 3 August Pi i Sunyer Biomedical Research Institute (IDIBAPS), 08036 Barcelona, Spain; 4 Biomedical Research Networking Center in Mental Health (CIBERSAM), 28029 Madrid, Spain; 5 Department of Fundamental and Clinical Nursing, Faculty of Nursing, University of Barcelona, 08907 Barcelona, Spain

**Keywords:** antipsychotics, biomarkers, cortisol, hair, illicit substances, neurotransmitters, schizophrenia, trace elements

## Abstract

Schizophrenia (SCZ) is a complex psychiatric disorder with positive, negative, and cognitive symptoms that cause long-term functional impairment. Despite available treatments, limitations in addressing cognitive and negative symptoms, medication side effects, and poor adherence highlight the need for novel biomarkers to support precision medicine. Hair has emerged as a promising, non-invasive biological matrix for biomarker research. Hair analysis has been explored to detect trace elements, abused drugs, antipsychotics, cortisol, neurotransmitters, and to assess gene expression, with results often consistent with blood, urine, and post-mortem tissues. Its advantages include ease of sampling and the ability to provide long-term information, while limitations involve individual variability and methodological inconsistencies. This review summarizes current evidence on hair-based biomarkers in SCZ, critically evaluating their potential and challenges. By integrating molecular, metabolic, and genetic findings, we highlight the value of hair as a complementary tool for advancing biomarker discovery and personalized approaches in SCZ.

## 1. Introduction

Schizophrenia (SCZ) is a severe mental illness with a lifetime prevalence of approximately 1%, typically developing in early adulthood [1]. Its diagnosis relies primarily on clinical assessment, often initiated when individuals with SCZ present positive symptoms, such as delusions and hallucinations. SCZ is also characterized by negative symptoms, such as amotivation and social withdrawal, and cognitive impairments, including deficits in working memory, executive functioning, and processing speed [2,3]. Notably, these prodromal symptoms often emerge during adolescence, highlighting this developmental stage as a critical window for early intervention to prevent long-term consequences of SCZ [4].

Given the complexity and chronicity of SCZ, early detection in individuals at risk is a major clinical and research priority. The prodromal phase, often emerging during adolescence, is characterized by subtle changes such as social withdrawal, emotional flattening, anxiety, and declining academic or occupational performance, features resembling negative symptoms but lacking full diagnostic specificity [5]. These early manifestations may represent the initial clinical expression of underlying neurobiological dysfunction and vulnerability, especially in individuals exposed to known risk factors. Among these, substance abuse, particularly cannabis use, has been identified as a significant modifiable factor that can accelerate the onset of psychosis and exacerbate its severity [6,7,8]. Numerous factors can also influence the course of the disease, including genetics, premorbid functioning, and adherence to treatment [5], as well as the individual’s psychosocial factors [9]. Importantly, accumulating evidence shows that early intervention during this window of vulnerability can significantly delay or even prevent the onset of psychosis, reduce symptom severity, and improve long-term functional outcomes [10,11].

This review aims to examine the current literature on the use of hair analysis and assess its potential applicability for biomarker detection in persons living with SCZ and those at risk. This biological matrix has demonstrated considerable promise due to its non-invasive nature and ability to provide retrospective data. We mainly structure this review by distinguishing between endogenous substances produced within the individual and exogenous biomarkers, which reflect environmental exposures, medications, or other external sources [12]. Given this context, we propose hair as a promising, non-invasive biological matrix for biomarker analysis, whose potential has been previously explored and supported in the literature [13,14,15].

### Hair Matrix as a Source of Potential Biomarkers in the Context of SCZ

In the context of SCZ, biomarkers are expected to aid in assessing the risk of developing the disorder, predicting the clinical course [16], and anticipating Antipsychotic (AP) side effects [17,18]. SCZ-related biomarkers encompass a broad spectrum, including metabolic, neurotransmitter-related, neurotrophic, systems-level, cellular, genetic, and epigenetic markers [19], among others. These can be categorized by their source, with central biomarkers derived from cerebrospinal fluid or post-mortem brains [20,21] and peripheral biomarkers obtained from biological matrices, such as hair [22], blood [20,23], or urine [24]. Currently, no single metabolite or biomarker panel has been demonstrated to reliably distinguish individuals with SCZ from healthy controls [25,26], likely reflecting the underlying clinical and biological heterogeneity of the disorder.

An emerging approach involves identifying specific subsets of individuals with SCZ or biotypes by stratifying them into more biologically homogeneous categories, a process that could enhance our understanding of the disorder’s neurobiological underpinnings. Within this framework, further research is needed to recognize robust and reproducible biomarkers capable of supporting this stratification. Nevertheless, several studies have demonstrated comparable proposed biomarker concentrations across various biological matrices, including hair, blood, serum, urine, cerebrospinal fluid, and brain tissue, highlighting hair as a promising candidate for such analyses [21,27,28].

**Figure 1:**
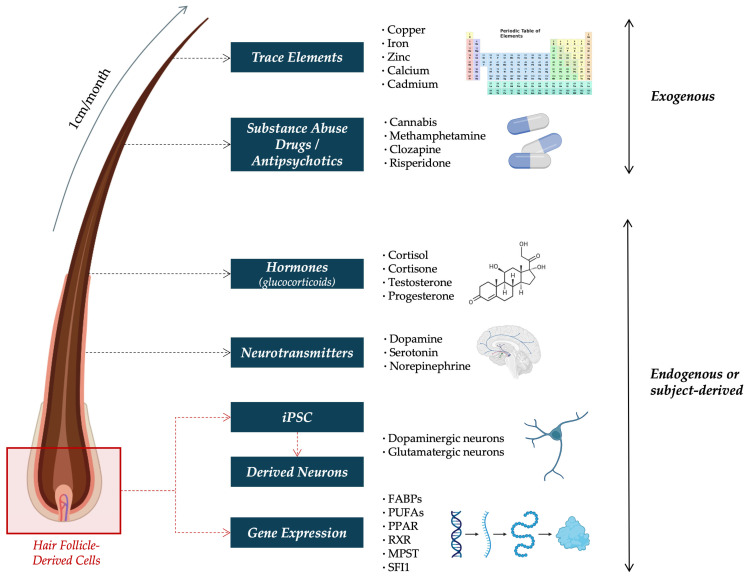
Schematic overview of exogenous and endogenous biomarkers detectable in hair in SCZ research. Exogenous compounds (e.g., trace elements, substance abuse drugs, and antipsychotics) reflect environmental exposure, while endogenous signals (e.g., hormones, neurotransmitters, gene expression, and subject-derived iPSCs) provide insight into physiological states. Abbreviations: FABP = Fatty Acid-Binding Protein; iPSC = Induced Pluripotent Stem Cells; MPST = Mercaptopyruvate Sulfurtransferase; PPAR = Peroxisome Proliferator-Activated Receptor; PUFA = Polyunsaturated Fatty Acid; RXR = Retinoid X Receptor; and SFI1 = Centrosome-Related Gene Stress Resistance Protein 1.

Hair grows at a relatively constant rate of 1 cm/month, making it a particularly suitable matrix for assessing long-term exposure to a wide range of substances [29]. The posterior vertex region close to the scalp is the preferred site for hair sampling, as it shows the least variability in growth rate and offers the most consistent results [30]. Depending on the length of the collected sample, exposure over periods ranging from weeks to several months can be evaluated [30]. For instance, analyzing the proximal 3 cm of hair can provide insight into endogenous or exogenous substance exposure over the previous three months [31,32]. Furthermore, segmental analysis, which involves dividing the hair into defined sections, enables the reconstruction of a retrospective timeline according to substance exposure or presence [29]. With this in mind, hair has been proposed as a promising matrix for assessing biomarkers relevant to SCZ, including trace elements [33], illicit drugs [34], hormone levels such as cortisol [35], neurotransmitters [36], and APs [37]. Additionally, emerging techniques have explored the use of scalp hair follicles as a source of gene expression for biomarker identification [14,38], as well as conducting Induced Pluripotent Stem Cells (iPSCs) and further differentiation into specific neuronal types, including dopaminergic and glutamatergic neurons [39], among others (Figure 1).

Hair has long been recognized as a promising and less invasive alternative or complementary matrix for biomarker identification, offering several advantages over traditional biological samples such as urine or blood. These conventional matrices are often limited by the short half-lives of many compounds [40,41], require invasive or time-sensitive collection procedures, and usually demand strict storage conditions to preserve the sample integrity [42,43]. In contrast, hair provides a non-intrusive biological record with the advantage of long-term storage without significant degradation [30,41,43]. Molecules incorporated into the hair shaft remain visible and traceable over time, enabling retrospective analysis [44]. Furthermore, it is well established that hair composition reflects blood constituents and their circulating concentrations [45,46]. Hair analysis has been successfully applied in SCZ [14,15] research and psychiatric disorders beyond SCZ, such as depression [47] and anxiety [48], as well as in other conditions, including cancer [49] and cardiovascular diseases [50].

The utilization of hair as a dependable instrument for the evaluation of biomarkers remains subject to certain limitations. Variables including age, sex, cosmetic hair treatments, and discrepancies in sampling and purification methodologies can affect the accuracy of biomarker detection [41,51]. In addition, results from hair analyses are often challenging to interpret, as the incorporation of substances into hair depends on multiple interacting variables, including hair growth rate, ethnicity, anatomical origin, substance metabolism, bioavailability, and interindividual variability [52]. Although the correlation between substance concentrations in hair and those in blood or urine is not always consistent or reliable [53], multiple studies have demonstrated a positive concordance between these biological matrices [27,28], thereby positioning hair analysis as a valuable potential avenue for future research.

## 2. Potential SCZ Biomarkers Derived from Hair

We classified the potential biomarkers of interest in SCZ according to their exogenous or endogenous origin. The selection of these molecules is grounded in the extensive literature, highlighting their relevance across key dimensions of SCZ, including core pathophysiological domains [54,55,56] as well as treatment-related factors [57,58,59] and environmental influences [60,61,62]. We performed a comprehensive literature search in PubMed and Scopus up to June 2025, using combinations of terms such as “schizophrenia”, “hair”, “biomarkers”, “trace elements”, “chemical compounds”, “antipsychotics”, “psychiatric medication”, “substance abuse”, “cannabis”, “methamphetamine”, “gene expression”, and “cellular models”. We prioritized original articles published in peer-reviewed journals, with particular attention to studies with human samples and clinical relevance.

### 2.1. Exogenous Biomarkers

#### 2.1.1. Trace Elements

An insufficiency in essential trace elements, such as calcium, iron, copper, zinc, cobalt, molybdenum, or selenium, may result in alterations in cognition, neurogenesis, memory, and learning [63,64], suggesting its contribution to the pathophysiology of various brain disorders [65], such as SCZ [56]. Therefore, hair analysis for trace elements emerges as a promising approach for estimating its long-term presence [66], potentially contributing to advancing knowledge in SCZ etiopathogenesis. Calcium has been shown to play a critical role in neuronal excitability, synaptic transmission, and plasticity. Alterations in intracellular calcium homeostasis have been documented in SCZ, both in several in vitro and post-mortem models [67,68], including changes in the expression of calcium channel subtypes. Genetic variants affecting calcium signaling have been linked with impaired calcium channel function, hippocampal atrophy, and cognitive symptoms in SCZ [69]. Furthermore, calcium-binding proteins, including calmodulin and parvalbumin, are altered in the cerebellum, hippocampus, and prefrontal cortex, among other brain regions, indicating dysregulated calcium signaling pathways in the disorder [70]. These disturbances may contribute to synaptic dysfunction, impaired neuroplasticity, and circuit-level abnormalities characteristic of SCZ.

Given the evidence of localized neuronal apoptosis observed in several SCZ models [71,72,73], the role of trace elements, particularly iron and calcium, in cell death mechanisms warrants attention. Iron is critically involved in ferroptosis, a distinct form of programmed cell death characterized by iron-dependent lipid peroxidation. Likewise, intracellular calcium also plays a central regulatory role in apoptosis. Taken together, the evidence of disrupted iron and calcium homeostasis, signaling, and redox imbalance in SCZ suggests that these trace element-related mechanisms may contribute significantly to the neurobiological underpinnings of the disorder.

A recent meta-analysis examining serum and plasma samples from persons diagnosed with SCZ and healthy controls revealed that individuals with SCZ exhibited altered trace element profiles. Specifically, copper levels were significantly elevated, whereas iron and zinc levels were significantly reduced [33]. These findings are consistent with the previous literature, which also reported correlations between specific trace elements in serum and metabolic parameters in persons living with SCZ, particularly those related to liver and renal function [56]. Additionally, abnormal iron accumulation has been reported in regions such as the prefrontal cortex and basal ganglia, both in vivo and post-mortem, suggesting lifelong dysregulation of iron homeostasis in individuals with SCZ [74,75]. These findings are consistent with those reported in the aforementioned meta-analysis [33]. Interestingly, iron is recognized as essential for brain development, particularly in energy metabolism, monoamine synthesis (especially dopamine), synaptogenesis, and myelination. Perinatal iron deficiency has been identified as an early risk factor for SCZ, potentially disrupting critical neurodevelopmental processes [76].

The use of hair trace element concentrations as biomarkers for long-term systemic alterations in SCZ [77,78,79] has been previously studied (Table 1). Complementing the serum and plasma findings, the literature reported that adolescents with psychosis-like symptoms had significantly lower hair zinc levels compared to those without such symptoms. Moreover, a negative correlation between zinc levels and psychosis risk was observed, which remained significant after adjusting for age and sex; however, no such association was found for copper levels [77]. Similarly, other studies have found that individuals with SCZ exhibited significantly decreased hair concentrations of iron, selenium, arsenic, potassium, manganese, and zinc, while copper, titanium, and cadmium levels were significantly higher compared to controls, with some of these differences varying by sex [78,79]. However, findings regarding calcium concentrations were inconsistent across studies [78,79]. Notably, one study reported a significant positive correlation between hair calcium levels and body mass index [78].

#### 2.1.2. Substance Abuse and Illicit Drugs

Hair analysis offers a valuable retrospective method for detecting substance use in individuals with SCZ, particularly given the role of illicit drugs in triggering and aggravating the disorder. Research suggests that certain individuals are more vulnerable to the psychotogenic effects of specific drugs, most notably cannabis and methamphetamines, in the development of substance-induced psychosis, which is strongly associated with an increased risk of developing SCZ [60,61,80]. Thus, the consumption of these illicit drugs can exacerbate psychotic symptoms and worsen the outcome of the disease [81,82]. It has been reported that comorbid addiction in individuals living with SCZ is associated with severe reductions in gray matter volume and executive functional deficits [83]. Additionally, the use of illicit substances in individuals with SCZ has been consistently linked with poorer adherence to pharmacotherapy, further compromising clinical outcomes [81,84].

Research also suggests that relying solely on patient self-reporting often leads to the significant underestimation of actual drug consumption [85]. For instance, individuals with SCZ have been shown to underreport drug consumption during acute psychotic episodes [86], as well as upon entry into early intervention programs for psychosis [87]. In this context, the relationship between substance use in SCZ [31,85,88] has been studied (Table 2). The literature revealed the significant underreporting of substance use among young individuals with SCZ, with 25% testing positive for amphetamines and 8% for cocaine, despite most denying use and their informants being unaware [88]. Underreporting substance abuse in persons living with SCZ was more common among women, African Americans, older individuals, and those with a longer illness duration or greater neurocognitive impairment, highlighting cognitive deficits as a strong and consistent predictor of inaccurate self-reporting [85]. Moreover, further research found that 27% of participants screened positive for cannabis, cocaine, or methamphetamine, with drug-positive individuals tending to be younger and presenting more severe positive symptoms, greater illness severity, and poorer premorbid indicators, such as childhood behavioral issues, while no overall differences across neurocognitive domains were observed [34]. Consequently, hair analysis has demonstrated significantly higher detection rates compared to both self-reports and urine testing, offering a more reliable and retrospective assessment of drug abuse [31,88,89], since urine toxicology assessment is constrained by the short detection window of many compounds [40,41] and the requirement for strict sample preservation conditions [42,43]. For example, a study on cocaine and cannabis hair assay demonstrated that cocaine use is detectable for 2–6 months after a single 25–35 mg dose of drug administration intravenously, while a positive urine test can only be expected within 2–3 days post-consumption [43]. This difference is relevant, as individuals may use their pharmacokinetic knowledge to temporarily stop substance use before testing, avoiding detection. Therefore, hair analysis serves as a valuable retrospective tool, offering a longer detection window and greater reliability.

Focusing on specific drug consumption, higher hair cannabinoid levels were associated with SCZ-like symptoms and organic brain dysfunction after 8–15 years of cannabis use, primarily observed in younger users who showed significant associations with hallucinations and delusions. Notably, memory impairment was also observed in all participants [90]. Interestingly, hair analysis revealed that individuals who only consumed delta-9 tetrahydrocannabinol (Δ9-THC) had higher scores on delusional and psychosis-like experiences than those consuming both Δ9-THC and Cannabidiol (CBD), suggesting the potential protective role of CBD against the psychotomimetic effects of Δ9-THC [91]. Regarding methamphetamine, higher hair concentrations have been reported in users with psychosis compared to non-users without psychosis, both groups having abstained for an average of 17 days before sampling, which correlated with reduced cognitive performance in addition to an increased risk of methamphetamine-induced psychosis [92]. Interestingly, further assays provided the first evidence of transplacental transfer of methamphetamine in humans, as evidenced by its accumulation in fetal hair at levels comparable to those observed in mothers [93]. These observations are particularly relevant in the context of SCZ, as they align with evidence linking prenatal methamphetamine exposure to an increased risk of developing the disorder later in life [62].

Conversely, an alternative report found inconsistencies between hair and urine testing, indicating discrepancies between approaches. However, combining hair and urine testing with self-reports significantly improved detection rates of substance use in individuals with SCZ, identifying 38% of users compared to only 16% through self-reporting alone [89]. Additionally, self-reported data, particularly among White populations, have demonstrated better concordance with the Structured Clinical Interview for the Diagnostic and Statistical Manual of Mental Disorders diagnoses, especially for cannabis, cocaine, and stimulant use. However, it remains unclear to what extent participants’ awareness of upcoming testing influenced the accuracy of their self-reported substance use. These findings suggest that, while biological matrices generally extend detection windows and reduce underreporting, self-reports may remain a valuable complement, especially when cultural or demographic factors improve their accuracy [31].

**Table 2:** Articles addressing substance abuse hair concentrations across people living with SCZ and associated risk populations.

First Author, Year	Sample	Method for Assessment	Main Results
Lamyai, W. (2019) [92]	Individuals with MAP (*n* = 113) and non-MAP (*n* = 120).	GC-MS	Significantly higher hair methamphetamine concentrations were present in users with psychosis, in addition to a reduced cognitive performance and an increased risk of MAP.
Nestoros, J.N. (2017) [90]	Cannabis users (*n* = 48).	GC-MS	Higher hair cannabinoid levels were associated with SCZ-like symptoms and organic brain dysfunction, particularly in younger users with significant hallucinations and delusions. Memory impairment was observed in all participants.
Bahorik, A.L. (2014) [85]	People living with SCZ (*n* = 1042).	RIA	Underreporting was significantly associated with older age and greater neurocognitive deficits, while accurate reporting was linked to more criminal justice involvement.
Bahorik, A.L. (2014) [34]	People living with SCZ + RIA (*n* = 262) and − RIA (*n* = 712).	RIA	Drug-positive individuals tended to be younger and presented highly severe positive symptoms, illness severity, and poorer premorbid indicators, such as childhood behavioral issues.
Van Dorn, R.A. (2012) [31]	People living with SCZ (*n* = 1460).	RIA	Hair testing resulted in the highest rates of over-detection compared to the SCID. Self-report data showed better concordance, accuracy, and agreement with SCID diagnoses within specific cultural or demographic situations.
Morgan, C.J.A. (2008) [91]	Individuals with Δ9-THC (*n* = 20), Δ9-THC + CBD (*n* = 27), and no cannabinoids (*n* = 85).	GC-MS	Individuals with only Δ9-THC detected in their hair showed significantly higher scores on positive symptoms, in addition to higher delusion scores compared with CBD and Δ9-THC + CBD group.
Garcia-Bournissen, F. (2007) [93]	Individuals positive for methamphetamine (babies; *n* = 19, children; *n* = 13, adults; and *n* = 247).	ELISA/GC-MS	Methamphetamine transplacental transfer was observed, as evidenced by accumulation in fetal hair at levels comparable to those observed in mothers.
Swartz, M.S. (2003) [89]	People living with SCZ (*n* = 201).	RIA	Hair testing showed higher time detection window compared to urine testing. Combining hair, urine, and self-reports significantly improved detection rates of substance use.
McPhillips, M.A. (1997) [88]	People living with SCZ (*n* = 36).	RIA/ELISA	Hair assays revealed significant underreporting of substance use among young individuals with SCZ, despite most denying use and their informants being unaware.

Summary: There is a complex interplay between SCZ and substance abuse, with hair analysis playing a significant role in uncovering drug use patterns [31,85,88]. While hair analysis offers a tool for objective measurement of drug use, its role in diagnosing or predicting outcomes in SCZ remains nuanced, with differing conclusions on its comparative accuracy and predictive power across various substances and symptomologies. In addition to studies involving individuals with SCZ, research on populations at elevated risk of psychosis has also been included (e.g., methamphetamine users, neonates exposed in utero), given the potential overlap in neurobiological mechanisms and the relevance for early biomarker identification. Abbreviations: CBD = Cannabidiol; ELISA = Enzyme-Linked Immunosorbent Assay; GC-MS = Gas Chromatography/Mass Spectrometry; MAP = Methamphetamine-Associated Psychosis; RIA = Radioimmunoassay; SCID = Structured Clinical Interview for The Diagnostic and Statistical Manual of Mental Disorders; SCZ = Schizophrenia; and Δ9-THC = Delta-9 tetrahydrocannabinol.

#### 2.1.3. Antipsychotics

Currently, no AP effectively addresses the totality of the three main symptomatic domains [94,95]. In addition, poor adherence to pharmacological treatment is prevalent in individuals with SCZ, driving an increasing risk of clinical worsening, adverse outcomes, and rehospitalization [96,97]. Moreover, the narrow therapeutic window of these treatments also contributes to increasing unpleasant side effects [37,98]. Therefore, therapeutic drug monitoring through hair has emerged as a promising alternative due to its non-invasive collection and extended detection window, potentially offering a longitudinal view of treatment exposure and adherence [99,100]. Although blood or plasma analysis is widely considered the gold standard for assessing AP adherence, particularly in cases of unclear compliance [59], nonresponse despite a sufficient dosage, or pronounced side effects despite a low dosage [101], conventional drug monitoring reflects only recent drug intake and may miss adherence fluctuations over longer periods.

Hair concentrations of certain APs can reflect long-term exposure and correlate with biological matrices [37,102,103] (Table 3). Recent studies have explored hair analysis in broader psychiatric populations and forensic contexts, revealing both its potential and inherent complexities. For instance, analysis of paired hair and nail samples from 13 individuals with SCZ undergoing chronic AP treatment, including clozapine, haloperidol, olanzapine, and quetiapine, showed that higher hair concentrations correlated with nail concentrations for haloperidol, levomepromazine, clozapine, and quetiapine; however, variable distribution patterns and extraction differences, particularly for olanzapine, highlighted potential limitations [104]. A comparable study examining 10 common APs simultaneously in blood, hair, and nails across individuals with various psychiatric disorders demonstrated significant overall correlations between blood and hair drug concentrations, supporting hair as a consistent matrix for reflecting drug exposure. Nonetheless, segmental hair analysis for olanzapine yielded false negatives, underscoring methodological constraints [105]. Additional research using risperidone demonstrated significant correlations between hair and serum concentrations, although dosage showed no statistically significant correlation [37]. Complementing these findings, albeit with a very limited sample size of *n* = 3, subsequent work identified a dose-concentration consistency in hair for risperidone, which was stronger for its metabolite 9-hydroxyrisperidone than for risperidone itself, indicating that metabolite analysis may enhance interpretability. Notably, correlations between hair concentration and AP dosage are not always observed in the literature [106].

With a particular focus on clozapine hair analysis, significant correlations between the daily dose and hair concentrations of clozapine, its metabolite norclozapine, and chlorpromazine have been reported, suggesting that, at least for these compounds, hair analysis could serve as a semi-quantitative biomarker of adherence [103]. This association is further supported by another clozapine hair study, where positive correlations between the daily clozapine dose and its concentration in 3 cm hair segments suggest that hair analysis can reliably reflect general dosing trends [102]. However, these studies also highlighted significant limitations and inconsistencies that constrain the clinical applicability of hair analysis. Notably, such discrepancies may arise from substantial interindividual variability and the influence of external factors, such as cosmetic treatments and hair color [37,103]. The melanin fraction of hair has been reported to bind APs more strongly than hair proteins, with large interindividual variability attributable to biochemical individuality [107].

### 2.2. Endogenous and Subject-Derived Biomarkers

#### 2.2.1. Cortisol

Cortisol, a key stress-related hormone, has been linked to SCZ, but traditional methods (blood, saliva, and urine) mostly capture short-term levels, limiting the accurate assessment of long-term secretion [108,109,110,111]. Individuals with SCZ are thought to exhibit an increased stress sensitivity and a heightened stress perception, influenced by environmental factors and the nature of the encountered burdens [32,35,109]. Although abnormal stress reactions have frequently been observed in people diagnosed with SCZ, the underlying causes and mechanisms remain incompletely understood. However, it is well established that the Hypothalamic–Pituitary–Adrenal (HPA) axis plays a central role in the regulation of the neuroendocrine stress response [32]. Dysfunction of the HPA axis may be associated with neurodevelopmental alterations in relevant brain regions such as the hippocampus [110]. Accordingly, elevated serum cortisol levels [55,111] have been described in individuals with SCZ, although inconsistent findings that have emerged from hair analysis have been reported [112,113,114]. The studies exploring Hair Cortisol Concentration (HCC) as a biomarker for long-term HPA axis activity in SCZ are summarized (Table 4). However, there is evidence suggesting that AP treatment may influence the HPA axis and therefore cortisol levels [32]. Studies examining HCC in SCZ report heterogeneous findings, with some showing elevations, others showing reductions, and some showing no significant changes. The following sections summarize this variability, highlighting key methodological and clinical factors that may account for such discrepancies. While the directionality of cortisol alterations varies across studies, the overall evidence suggests a dysregulation of the HPA axis.

On the one hand, lower HCC has been reported in individuals with SCZ compared to healthy controls, even when exposed to high levels of psychosocial stress [115,116]. Therefore, the inverse relationship with psychosocial factors may imply an adaptive dysfunction of the HPA axis in SCZ, also known as hypocortical blunting or resistance to chronic stress. Typically, elevated HCC is generally observed in individuals under persistent stress situations unrelated to SCZ, yet certain psychiatric conditions, such as post-traumatic stress disorder, tend to exhibit a reduction in HCC, highlighting distinct patterns of HPA axis activity across diagnoses [112]. In line with this complexity, research observed a negative association between HCC and the severity of SCZ’s psychotic symptoms. Their findings also indicated that persons diagnosed with SCZ reported significantly more childhood adversities than controls using the Maltreatment and Abuse Chronology of Exposure scale, with early abuse experiences predicting lower HCC levels [117]. Similarly, further research found significantly reduced baseline HCC in individuals with SCZ compared to controls, suggesting a blunted HPA axis and decreased long-term cortisol output. Notably, this pattern was not observed in First-Episode Psychosis (FEP) individuals, whose HCC levels increased over a 12-month period, and in relation to metabolic syndromes, potentially reflecting early-stage HPA axis dysregulation [118].

On the other hand, significantly higher levels of HCC were observed both in drug-naïve FEP individuals and in SCZ pregnant women, the latter also presenting with poorer symptomatic functioning [119,120]. Additional studies have further highlighted endocrine dysregulation in SCZ, as significant endocrine alterations in individuals with SCZ have been reported, including a markedly altered cortisol-to-cortisone ratio, reduced testosterone and progesterone (especially in females), and an elevated cortisol/testosterone ratio. These findings suggest dysregulation of both the HPA and the hypothalamic–pituitary–gonadal axes, possibly contributing to an increased vulnerability to stress-related pathophysiology in SCZ [35]. Subsequent work reported elevated HCC in persons diagnosed with SCZ with a history of childhood maltreatment using the Childhood Trauma Questionnaire [109]. Supporting this link, additional research observed a significant association between elevated HCC and exposure to traumatic events, particularly childhood abuse [121]. A related exploration highlighted a substantial genetic contribution to the variance in HCC and related psychological traits, emphasizing the heritability of stress-related endocrine profiles [122].

However, the literature also evidences several investigations that have reported no significant differences in HCC between individuals with SCZ and healthy controls [123,124]. For instance, one study found that although perceived stress was elevated in persons living with SCZ, HCC did not differ significantly from that of control participants, suggesting a potential dissociation between subjective stress perception and long-term cortisol output [123]. These observations align with meta-analytic evidence, where no significant group differences in HCC were observed, highlighting the substantial heterogeneity across studies and suggesting that normalized HCC levels may reflect the influence of AP medication or therapeutic interventions on long-term HPA axis regulation in individuals with SCZ [32].

A major limitation in studying HCC in SCZ lies in the high inter- and intra-individual variability in cortisol levels and the known sensitivity of cortisol measurements to methodological differences. Contradictory findings may arise from methodological differences, variations in patient characteristics, and the influence of medication.

**Table 4:** Articles addressing HCC across people living with SCZ and associated risk populations.

Authors, Year	Sample	Method for Assessment	Main Results
Qi, D. (2024) [35]	People living with SCZ (*n* = 137) and healthy controls (*n* = 73).	LC-MS/MS	SCZ individuals showed markedly altered cortisol-to-cortisone ratio, reduced testosterone and progesterone (specially in females), and an elevated cortisol/testosterone ratio.
Nyström-Hansen, M. (2024) [119]	Pregnant women in the 3rd trimester with SCZ, BP, or depression (*n* = 32) and healthy controls (*n* = 37).	LC-MS/MS	Both lifetime diagnosis of serious mental illness and poorer current symptomatic functioning were significantly associated with increased HCC in pregnancy.
Brandt, J.M. (2023) [124]	Children with at least one parent with a register-based diagnosis of SCZ (*n* = 111), BP (*n* = 82), and healthy controls (*n* = 129).	CLIA	HCC did not differ significantly across the three groups. Higher levels of perceived stress were not associated with higher HCC. Children at familial high-risk of SCZ reported higher perceived stress compared to controls.
Van Den Heuvel, L.L. (2022) [118]	People living with SCZ (*n* = 16) and healthy controls (*n* = 21).	LC-MS/MS	At baseline, HCC was significantly lower in people diagnosed with SCZ. HCC increased from baseline to month 12 in FEP individuals, demonstrating a trend towards significance and in relation to metabolic syndrome.
Yang, F. (2021) [115]	People living with SCZ (*n* = 109), BP (*n* = 93), and healthy controls (*n* = 86).	ELISA	HCC was significantly lower in people diagnosed with SCZ, also associated with clinical symptoms. HCC related positively with social support and personality traits, suggesting complex HPA axis involvement.
Yang, F. (2020) [116]	People living with SCZ (*n* = 109) and healthy controls (*n* = 86).	ELISA	People diagnosed with SCZ showed significantly lower HCC than controls despite higher psychosocial stress. HCC negatively correlated with delusions and trended with tension and uncooperativeness.
Söder, E. (2019) [121]	Familial risk group for psychosis (*n* = 32), clinical risk group for psychosis (*n* = 43), and low-risk controls (*n* = 35).	CLIA	HCC did not differ between individuals at clinical or familial risk and low-risk controls. However, HCC was significantly associated with exposure to traumatic events and childhood abuse.
Aas, M. (2019) [109]	People living with SCZ (*n* = 28), BP (*n* = 35), and healthy controls (*n* = 94).	ELISA	HCC was highest in people diagnosed with SCZ and showed greater variance than in controls. People diagnosed with SCZ with a history of childhood maltreatment also exhibited elevated HCC.
Hirt, V. (2019) [117]	Individuals at risk for psychosis (*n* = 29), people living with early SCZ (*n* = 34), people living with chronic SCZ (*n* = 24), and healthy controls (*n* = 38).	CLIA	A negative association between HCC and the severity of SCZ’s psychotic symptoms was observed. SCZ individuals reported significantly more childhood adversities within predicting lower HCC levels.
Rietschel, L. (2016) [122]	Individuals including 8 monozygotic, 21 dizygotic twin pairs, and 51 unpaired twins (*n* = 109).	CLIA	HCC was associated with psychological factors, along with substantial genetic contributions to the variance in HCC, emphasizing the heritability of stress-related endocrine profiles.
Streit, F. (2016) [123]	People living with SCZ (*n* = 159), BP (*n* = 61), and healthy controls (*n* = 82).	LC-MS/MS	While perceived stress is elevated in SCZ, the HPA axis activity, as measured by HCC, did not significantly differ from controls.
Andrade, E.H. (2016) [120]	Drug-naïve individuals with FEP (*n* = 24) and healthy controls (*n* = 27).	ELISA	HCC increased in drug-naïve FEP individuals. Moreover, differences in hair cortisol between segments representing different time points were correlated with the severity of psychopathology.

Summary: The literature reveals considerable variability in HCC findings among individuals with SCZ, reflecting the complex interplay between stress, HPA axis regulation, and illness characteristics [125]. While several studies report reduced HCC in persons living with SCZ [115,116], others show elevated HCC, particularly in subgroups with FEP or histories of trauma and childhood adversity [109,120]. Additionally, some studies report no significant differences [32,123,124]. Factors such as childhood trauma, stressful life events, and social support appear to have an inverse relationship with cortisol in individuals living with SCZ. Abbreviations: BP = Bipolar Disorder; CLIA = Chemiluminescence Immunoassay; ELISA = Enzyme-Linked Immunosorbent Assay; FEP = First-Episode Psychosis; HCC = Hair Cortisol Concentration; HPA = hypothalamic–pituitary–adrenal; LC-MS/MS = Liquid Chromatography-Mass Spectrometry; and SCZ = Schizophrenia.

#### 2.2.2. Neurotransmitters

SCZ has been associated with disruptions in several neurotransmitter systems, including dopamine, glutamate, and serotonin. Interactions among these alterations highlight the importance of fronto-thalamo-striatal–midbrain circuits in the pathophysiology of the disorder [1,126]. Therefore, analyzing these chemical compounds may enhance our understanding of neurotransmitter dysregulation and aid in the identification of potential biomarkers [127].

To date, only one study has reported evidence regarding SCZ and hair analysis (Table 5), in addition to a few observations in blood and urine. For instance, a recent study (2024) found that Liquid Chromatography-Mass Spectrometry (LC-MS/MS) was accurate in determining the endogenous levels of neurotransmitters, including dopamine, serotonin, and norepinephrine, in real human blood and urine samples from healthy individuals. These results demonstrated the reliability of the method, even with small sample quantities [128], thereby laying the groundwork for future and expanded research in hair samples. A study conducted in an animal model (red deer) demonstrated that the LC-MS/MS methodology developed was capable of determining dopamine and other catecholamines in both urine and hair samples [36]. Notably, just one study developed a sensitive Gas Chromatography/Mass Spectrometry method to measure tyramine in hair samples from individuals with SCZ. This investigation demonstrated significantly higher tyramine levels in persons diagnosed with SCZ compared to healthy controls. As a trace amine derived from tyrosine, tyramine has been implicated in affective and cognitive dysfunction, suggesting its potential utility as a biomarker in SCZ research [129].

#### 2.2.3. Genetics, Transcriptomics, iPSC, and Derived Neurons

Hair follicle scalp cells have proven valuable due to their high colony-forming efficiency [130] and reliable DNA and RNA extraction, offering a novel, minimally invasive source of patient-derived genetic material, owing to their shared embryonic developmental origin [14] also in the context of SCZ (Table 6). Investigations identified Fatty Acid-Binding Protein 4 (FABP4) mRNA in hair follicles as a potential biomarker for SCZ, related to the GABAergic system, myelination, and fatty acid metabolism, with consistent and significant downregulation (around 40–43%). With a sensitivity of 71.8% and specificity of 66.7%, FABP4 expression may represent another promising, non-invasive biomarker, supporting further research into its role in disease pathogenesis [14]. Analyses have also consistently revealed a reduced expression of nuclear receptor genes such as the Retinoid X Receptor Alpha (RXRA) and Peroxisome Proliferator-Activated Receptor Alpha (PPARA), reflecting alterations previously reported in post-mortem SCZ brain tissue [46]. Supporting the link between diet, epigenetic regulation, and brain function, persons living with SCZ have demonstrated a significantly reduced expression of RXRA, PPARA, and PPARB/D in hair follicle cells [38]. In parallel, additional findings have reported the upregulation of Mercaptopyruvate Sulfurtransferase (MPST) expression in hair follicles from a subgroup of people with SCZ with sulfide stress, mirroring molecular changes observed in the brain [131]. Additionally, the decreased expression of other disease-relevant genes, including the Centrosome-Related Gene Stress Resistance Protein 1 (SFI1), has been observed, further supporting the potential of hair follicles as translational biomarkers of disease-associated molecular alterations [132].

Alongside genetic analyses, iPSCs derived from hair follicles and derived neuronal cell types have been used to model neurodevelopmental abnormalities in SCZ [39,133]. This approach provides a dynamic platform to study the functional consequences of genetic variants and epigenetic changes. For instance, an iPSC study found that SCZ-derived hair follicle cells exhibit profound dopaminergic differentiation defects, including abnormal morphology, reduced neurite outgrowth, absence of mature markers such as dopamine transporters, and decreased dopamine release [39]. Similarly, patient-derived glutamatergic and dopaminergic neurons showed immature phenotypes, with the absent expression of critical maturation markers (e.g., Tbr1), fewer synaptic contacts, and disrupted glutamate–glutamine cycling. Moreover, mitochondrial function was compromised, with impaired respiration, altered membrane potential, and reduced network connectivity, especially during dopaminergic differentiation [39]. Although this approach has not yet been proven superior to existing methods, it represents an underexplored methodological avenue. Therefore, its potential remains substantial and warrants further investigation.

## 3. Discussion

This review aimed to examine the current literature on the use of hair analysis and assess its potential applicability for biomarker detection in individuals with SCZ and those at risk. The selection of the mentioned biomarkers discussed above was grounded in the extensive literature that underscores their relevance across key dimensions of SCZ, including core pathophysiological domains [54,55,56], as well as treatment-related factors [57,58,59] and environmental influences [60,61,62]. Notably, alterations in many of these biomarkers show consistency with previous findings in brain tissue, cerebrospinal fluid, blood, and urine, further supporting their potential applicability in the context of hair-based analysis in SCZ (Table 7). While Table 7 highlights convergent findings across the hair, brain, CSF, blood, and urine, it also illustrates important inconsistencies. For instance, trace element levels such as zinc, iron, or manganese show opposite directions of change depending on the biological matrix or study design. Similarly, cortisol and glutamate present heterogeneous results across different tissues. These discrepancies likely reflect methodological heterogeneity, small sample sizes, and population differences, but they may also indicate that distinct biological matrices capture different temporal or pathophysiological dimensions of SCZ. Acknowledging these inconsistencies is essential for interpreting findings critically and for designing future studies that use standardized protocols and multimodal approaches. To facilitate comparison across biological matrices, we have summarized the main advantages and disadvantages of hair relative to the brain, CSF, blood, and urine (Table 8). To date, no single biomarker or validated panel exists for the definitive identification of SCZ since it is a complex and heterogeneous disorder, characterized by diverse underlying pathological mechanisms and requiring multidisciplinary approaches for both the investigation and clinical management [134]. The identification of reliable biomarkers could facilitate the stratification of individuals diagnosed with SCZ into more biologically homogeneous subgroups or biotypes, thereby improving our understanding of the disorder’s neurobiological underpinnings and informing more targeted therapeutic strategies [6,134].

This review focuses on the key biomarkers of potential relevance in the context of SCZ, with the rationale for their inclusion outlined as follows: (i) trace elements are essential for cognition, neurogenesis, memory, and learning [63,64]. (ii) Illicit substances contribute to the development of substance-induced psychosis, which is strongly associated with an increased risk of transitioning to SCZ [60,80]. (iii) AP treatment is frequently associated with poor adherence, which increases the risk of clinical deterioration, adverse outcomes, and rehospitalization [96,97]. (iv) Cortisol dysregulation is also considered a key factor mediating the relationship between chronic stress and the onset or progression of SCZ [120,135]. (v) Neurotransmitters are fundamental to the interplay between the nervous system and other physiological systems [136,137], and deficiencies in these compounds underlie rare neurological disorders with early clinical manifestations [138]. (vi) Hair follicles and their gene expression profiles have been explored in several studies as a source for biomarker identification in SCZ [14,38], also showing the potential for reprogramming into iPSCs and subsequent differentiation into dopaminergic and glutamatergic neurons [39]. Altogether, the features of these biomarkers could underscore the multidimensional nature of SCZ and position hair as a valuable matrix for capturing this complexity and advancing biomarker-driven research in the field.

For each of these selected biomarkers, we briefly describe below the hair-related findings in connection with SCZ. (i) Several studies have reported altered trace element levels in the hair of individuals with SCZ, including decreased zinc and calcium and increased copper and cadmium. These imbalances appear in both adolescents at risk and adults with SCZ, suggesting a potential role in the disorder’s pathophysiology [77,78,79]. (ii) Hair analysis consistently revealed underreported substance use among individuals with SCZ, particularly for cannabis, cocaine, and amphetamines. It has proven to be a reliable and non-invasive tool for detecting drug exposure, often outperforming self-reports and complementing other biological measures [31,34,85,88,89,90,91,92,93]. (iii) Studies show variable correlations between the AP dose and hair concentration, with stronger associations for clozapine and its metabolite, but high interindividual variability and factors such as adherence and melanin-binding limit the reliability of hair for therapeutic monitoring [37,102,103,104,105,106,107]. (iv) HCC in SCZ shows inconsistent results: while some studies report lower [115,116,117,118] or higher [35,109,119,120,121,122] levels compared to controls, others find no significant differences [32,123,124]. Variability is influenced by factors such as stress, trauma, psychopathology severity, sex, and treatment. Overall, HCC reflects complex and heterogeneous HPA axis dynamics in SCZ, pointing to alterations but limiting its utility as a standalone biomarker. (v) Hair tyramine levels were significantly altered in individuals with SCZ, suggesting its potential as a biomarker [129]. (vi) Gene expression analyses and stem cell generation from the hair follicle cells of individuals with SCZ revealed the dysregulation of nuclear receptors, fatty acid metabolism, and neuronal differentiation pathways, supporting their potential as non-invasive biomarkers linked to core pathophysiological mechanisms [14,38,39,46,131,132,139].

Hair has long been recognized as a promising alternative for biomarker identification, offering several advantages over traditional biological samples such as urine and blood. It provides a non-invasive method for sample collection and allows for long-term storage without significant degradation [30,41,43]. Moreover, the integration of compounds into the hair shaft enables retrospective analysis, as incorporated molecules remain stable and traceable along the hair length over time [44]. Therefore, hair analysis offers notable clinical benefits with a longer detection window [43], making it less susceptible to manipulation. Furthermore, hair also shows promise in pharmacogenetic and drug monitoring applications [59], such as identifying genetic polymorphisms that influence drug metabolism and treatment response. Genetic studies using hair-derived DNA have yielded consistent and reliable results [38,139], underscoring its potential as a non-invasive source of genetic material for precision medicine approaches.

However, hair analysis faces significant limitations. These include methodological inconsistencies for compound identification across studies, the influence of cosmetic treatments, variability in hair type, or insufficient reporting, across populations. Additionally, many studies often present limited statistical power due to small sample sizes or the lack of control groups. Indeed, there is currently no standardized protocol universally adopted in the field, and many studies do not systematically report or control for cosmetic hair treatments (e.g., bleaching, dyeing) or other chemical processes known to bias drug incorporation and detection [52,140,141]. Additional sources of variability include differences in hair color, melanin content, and individual physiological or behavioral factors that can affect analyte distribution [140]. Taken together, these challenges underscore the need for standardized protocols that account for population-specific hair types, thereby enabling the validation and broader implementation of hair-based biomarker identification models. There is an urgent need for further investigation into novel hair-based biomarkers relevant to SCZ, as current evidence remains limited and fragmented, especially considering the current lack of robust evidence in many domains. Advancing this field will require not only the identification of new biomarkers but also careful consideration of previously examined biomarkers and the discussed sources of variability, including methodological inconsistencies, hair type, and external influences such as cosmetic treatments.

Despite its advantages, hair analysis remains underutilized in SCZ research. Compared to other common medical conditions, there is a marked shortage of studies examining hair-based biomarkers in individuals with SCZ. In contrast, hair analysis has been successfully applied in research on cancer [49], cardiovascular diseases [50], and psychiatric conditions including depression [47], anxiety [48], and autism spectrum disorder, where altered amino acid profiles (e.g., decreased methionine, alanine, and asparagine; increased glutamic acid) have been observed [142]. Key domains of SCZ pathophysiology, such as neurotransmitter levels and epigenetic modifications, remain largely unexplored through this matrix despite their potential to offer critical insights into the disorder. This underrepresentation suggests that SCZ remains a relatively neglected field in biomarker research despite its clinical complexity and public health impact, highlighting the need for greater investment and focus in this area.

**Table 7:** Summary of representative biomarker findings across the brain, CSF, blood, urine, and hair in the context of SCZ. This table provides a representative overview of current findings across various biological matrices that align with those observed in hair analysis. While it offers an illustrative synthesis of consistent results, it is intended as a reference tool.

Biomarker		Hair	Brain	CSF	Blood	Urine
Trace Elements	Copper	↑ [78,79]	↓ [143,144,145]	↓ [146]	↑ [33,145,147]	↑ [148]
	Zinc	↓ [77,78,79]	↑/x [149,150,151]	x [152]	↓ [33,149,150]	↓ [148]
	Iron	↓ [79]	↑↓ [74,75]	↓ [153]	↓ [33,147,154,155,156]	-
	Calcium	↓ [78,79]	↓ [157,158]	↑ [159]	x [160]	↓ [161]
	Cadmium	↑ [78]	↑ [162]	-	↑ [163,164]	-
	Manganese	↓/x [78,79]	↑ [165]	-	↑↓ [56,147,151,166,167,168,169]	↑ [170]
Substance Abuse Drugs		↑ [34,85,90,91,92]	↑ [171,172]	↑ [173]	↑ [174]	↑ [88,175,176]
Antipsychotics		↑ [99,100]	↑ [177,178,179]	↑ [178,179,180]	↑ [59,101,179]	↑ [57,180,181]
Cortisol		↑↓ [115,116,120]	↑ [181,182]	↑ [183]	↑ [54,113,184]	↑ [185,186]
Neurotransmitters	Dopamine	-	↑ [187,188,189,190]	↑ [191]	↑ [192,193]	↑ [194,195]
	Serotonin	-	↑ [196]	↑/x [197,198,199]	↑ [200,201]	↑ [202]
	Glutamate	-	↑↓ [203,204]	↓ [203,205]	↑↓ [206,207]	↑ [208]
	Tyramine	↑ [129]	-	-	↓ [209]	↓ [210]
Genetics and Transcriptomics	FABP	↓ [14]	↓ [211]	-	-	-
	RXR	↓ [38,46]	↓ [38,46]	-	↓ [46]	-
	PPAR	↓ [38,46]	↓ [38,46]	-	↓ [212]	-
	MPST	↑ [131]	↑ [131]	-	-	-
	SFI1	↓ [132]	-	-	-	-

Arrows indicate the direction of biomarker alterations in individuals with SCZ: ↑ = increased levels, ↓ = decreased levels, ↑↓ = mixed or inconsistent findings, x = no significant differences observed, and - = data not available. Abbreviations: CSF = cerebrospinal fluid, FABP = Fatty Acid-Binding Protein, MPST = Mercaptopyruvate Sulfurtransferase, PPAR = Peroxisome Proliferator-Activated Receptor, RXR = Retinoid X Receptor, SCZ = Schizophrenia, and SFI1 = Stress Resistance Protein 1.

**Table 8:** Advantages and disadvantages of different biological matrices for biomarker research in SCZ.

Matrix	Hair	Brain Tissue	CSF	Blood	Urine
Advantages	Non-invasive, painless; retrospective long-term analysis; stable storage; detects chronic exposure (drugs, hormones, and trace elements); and potential for gene expression and iPSC reprogram-ming	Direct assessment of central pathophysiology; allows regional analysis; and gold standard for molecular/structural analyses	Reflects CNS biochemistry directly; useful for neurotransmitters and inflammatory markers	Widely used; standardized; minimally invasive; repeated sampling; and good for systemic biomarkers	Non-invasive; easy large-volume collection; and suitable for metabolic and drug screening
Disadvantages	Affected by cosmetic treatments; potential external contamination; variability by hair type/color/melanin; no standardized protocols; and limited SCZ studies	Invasive; post-mortem or rare biopsy only; influenced by agonal state; limited sample size; and not longitudinal	Invasive lumbar puncture; low acceptability; small volume; and potential complications	May not fully reflect brain-specific processes; temporal fluctuations; and some analytes unstable	Rapid turnover; high intraindividual variability; influenced by hydration/diet; and less relevant to CNS

Abbreviations: CNS = central nervous system, iPSC = Induced Pluripotent Stem Cells, and SCZ = Schizophrenia.

## 4. Conclusions

Hair analysis emerges as a promising, non-invasive tool for the detection of a wide range of exogenous and endogenous biomarkers relevant to SCZ. Current evidence supports its utility for assessing trace elements, illicit substances, AP exposure, cortisol, neurotransmitters, gene expression signatures, and neurodevelopment. Despite its methodological viability and concordance with traditional matrices, the scarcity of SCZ-specific studies and the lack of standardized protocols currently limit its broader application. Expanding research efforts and establishing methodological consensus are essential steps to validate hair-based biomarkers and unlock their potential for advancing diagnosis, treatment monitoring, and pathophysiological insight in SCZ.

## Figures and Tables

**Table 1:** Articles addressing trace elements of hair concentrations across people living with SCZ and associated risk populations.

First Author, Year	Sample	Method for Assessment	Main Results
Pradeep, A.S. (2023) [79]	People living with SCZ (*n* = 60) and healthy controls (*n* = 30).	PIXIE	Decreased hair concentrations of iron, selenium, arsenic, potassium, manganese, and zinc were observed, while copper, titanium, and calcium levels were significantly higher compared to controls. Differences appeared to vary by sex.
Tabata, K. (2022) [77]	Drug-naïve adolescents with psychosis risk (*n* = 252).	ICP-MS	Adolescents with psychosis-like symptoms had significantly lower hair zinc levels compared to those without symptoms, suggesting its involvement in the pathophysiology of psychosis.
Rahman, A. (2009) [78]	People living with SCZ (*n* = 30) and healthy controls (*n* = 30).	FAAS	Hair concentrations of zinc and calcium were significantly decreased, while copper and cadmium levels were significantly elevated compared to controls, with no change in manganese levels.

Summary: The combined findings from hair and serum analysis suggest a consistent association between trace element imbalances and SCZ. Iron and calcium are not only essential for neurodevelopment but are also critically involved in mechanisms of programmed cell death, suggesting their involvement in neuronal dysfunction and the broader pathophysiology of the disorder [70,71,72,73,76]. Observed alterations may reflect underlying metabolic or physiological disruptions linked to the disorder and highlight the potential of trace elements as biomarkers in SCZ research. Abbreviations: FAAS = Flame Atomic Absorption Spectroscopy; ICP-MS = Inductively Coupled Plasma Mass Spectrometry; PIXIE = Particle-Induced X-Ray Emission; and SCZ = Schizophrenia.

**Table 3:** Articles addressing AP hair concentrations across people living with SCZ, associated risk populations, or under AP treatment.

First Author, Year	Sample	Method for Assessment	Main Results
Yang, H. (2023) [105]	Individuals with psychiatric disorders (*n* = 54) and forensic suspects samples (*n* = 2).	LC-MS/MS	Significant correlations were found between blood and hair concentrations. Segmental hair analysis revealed false negatives for olanzapine, highlighting potential forensic limitations.
Cobo-Golpe, M. (2020) [104]	People under chronic AP treatment (*n* = 13).	LC-MS/MS	Higher hair APs concentrations correlated with nail concentrations, with distribution patterns varying across drugs. Inconsistent patterns were observed for olanzapine, influenced by extraction variability and limited case numbers.
Ramírez-Fernández, M. (2020) [107]	Individuals under AP treatment (*n* = 59).	UHPLC-MS/MS	No clear correlation between prescribed dose and hair concentration was observed. Results showed that melanin fraction of hair bonded AP more strongly than hair proteins.
Wang, X. (2019) [103]	People living with SCZ (*n* = 46).	LC-MS/MS	For CLZ, norclozapine, and chlorpromazine, a significant relationship was found between dose and hair segments, suggesting hair analysis as a semi-quantitative biomarker of adherence.
Sun, X. (2019) [37]	People living with SCZ treated with RSP for more than 3 months (*n* = 34).	LC-MS/MS	Significant correlation between hair concentration of RSP with its serum concentration was observed. The dosage had no statistically significant correlation with the hair concentration of RSP.
Schneider, S. (2009) [106]	People living with SCZ (*n* = 1), psychosis (*n* = 1), and autism (*n* = 1).	LC-MS/MS	Significant correlations between RSP concentrations in hair and serum were observed, supporting the notion that hair concentrations can mirror systemic exposure over time.
Cirimele, V. (2000) [102]	People living with SCZ treated with CLZ (*n* = 26).	GC-MS	A better dose–concentration relationship of CLZ was observed between daily dose and hair concentration but with wide variations in the SCZ cohort.

Summary: While serum measurements remain the gold standard for detecting AP dosage variations [59,101], several studies demonstrate that hair analyses are technically feasible and capable of qualitatively confirming chronic AP use. Of note, this approach has limitations in quantitative interpretation. Abbreviations: AP = Antipsychotic; CLZ = clozapine; GC-MS = Gas Chromatography/Mass Spectrometry; LC-MS/MS = Liquid Chromatography-Mass Spectrometry; RSP = risperidone; SCZ = Schizophrenia; and UHPLC-MS/MS = Ultra-High Performance Liquid Chromatography-Tandem Mass Spectrometry System.

**Table 5:** Study addressing neurotransmitter findings in SCZ.

Authors, Year	Sample	Method for Assessment	Main Results
Gao, L. (2016) [129]	People living with SCZ (*n* = 95), individuals who abuse drugs (*n* = 56), and healthy controls (*n* = 90).	GC-MS	Significant difference in hair tyramine levels between people with SCZ and healthy controls were observed. Sex difference was not statistically significant. These findings suggest that tyramine may serve as a potential biomarker for SCZ and merits further investigation.

Summary: Current evidence supports the feasibility of using hair for neurotransmitter and amino acid analysis, offering a minimally invasive approach that may complement classical blood or urine biomarkers in psychiatric research [128]. However, despite its methodological viability, the number of studies addressing this application remains limited in SCZ, with only one identified to date. Abbreviations: GC-MS = Gas Chromatography/Mass Spectrometry; SCZ = Schizophrenia.

**Table 6:** Articles addressing genetic, iPSCs, and derived neuron findings in people living with SCZ and associated risk populations.

Authors, Year	Sample	Method for Assessment	Main Results
Wada W. (2020) [46]	People living with SCZ (*n* = 1) and healthy controls (*n* = 3).	RT-PCR	Hair follicle cells from people diagnosed with SCZ showed reduced expression of nuclear receptor genes RXRA and PPARA, also observed in postmortem SCZ brains.
Ide, M. (2019) [131]	People living with SCZ (*n* = 149) and healthy controls (*n* = 166).	IHC/ISH	MPST mRNA expression was significantly increased in hair follicle cells from a subgroup of persons diagnosed with SCZ, mirroring upregulation observed in brain tissue.
Matsuura, A. (2018) [132]	People living with SCZ (*n* = 94) and healthy controls (*n* = 117).	RT-qPCR	SFI1 mRNA expression was significantly reduced in hair follicle cells from people diagnosed with SCZ, consistent with protein-level changes in postmortem SCZ brains.
Maekawa, M. (2017) [38]	2 sample sets of people living with SCZ (1: *n* = 52; 2: *n* = 42) and healthy controls (1: *n* = 62; 2: *n* = 55).	RT-qPCR	People diagnosed with SCZ exhibited significantly reduced expression of RXRA, PPARA, and PPARB/D in hair follicle cells. Nuclear receptor dysregulation due to PUFA deficiency could be a key upstream event in SCZ pathophysiology.
Maekawa, M. (2015) [14]	2 sample sets of people living with SCZ (1: *n* = 52; 2: *n* = 42) and healthy controls (1: *n* = 62; 2: *n* = 55).	RT-qPCR	FABP4 mRNA was demonstrated as a potential genetic biomarker for SCZ, showing consistent and significant downregulation.
Robicsek, O. (2013) [39]	People living with SCZ (*n* = 3) and healthy controls (*n* = 2).	HPLC	Glutamatergic and dopaminergic neurons showed immature phenotypes. Moreover, mitochondrial function was compromised, with impaired respiration, altered membrane potential, and reduced network connectivity.

Summary: iPSC-derived neurons from hair of individuals with SCZ display significant impairments in differentiation, synaptic maturation, and mitochondrial function [39], offering a dynamic platform for studying disease mechanisms and identifying potential therapeutic targets. Abbreviations: FABP4 = Fatty Acid-Binding Protein 4; HPLC = High-Performance Liquid Chromatography; IHC = Immunohistochemistry; ISH = In Situ Hybridization; MPST = Mercaptopyruvate Sulfurtransferase; mRNA = Messenger RNA; PPARA = Peroxisome Proliferator-Activated Receptor Alpha; PPARB/D = Peroxisome Proliferator-Activated Receptor Delta; PUFA = Polyunsaturated Fatty Acid; RT-qPCR = Real-Time Quantitative Reverse Transcription PCR; RXRA = Retinoid X Receptor Alpha; SCZ = Schizophrenia; and SFI1 = Stress Resistance Protein 1.

## Abbreviations

The following abbreviations are used in this manuscript:

AP	Antipsychotic
BP	Bipolar Disorder
CBD	Cannabidiol
CLIA	Chemiluminescence Immunoassay
CLZ	Clozapine
CSF	Cerebrospinal Fluid
ELISA	Enzyme-Linked Immunosorbent Assay
FAAS	Flame Atomic Absorption Spectroscopy
FABP	Fatty Acid Binding Protein
FEP	First-Episode Psychosis
GC-MS	Gas Chromatography/Mass Spectrometry
HCC	Hair Cortisol Concentration
HPA	Hypothalamus-Pituitary-Adrenal
HPLC	High-Performance Liquid Chromatography
ICP-MS	Inductively Coupled Plasma Mass Spectrometry
IHC	Immunohistochemistry
ISH	In Situ Hybridization
iPSCs	Induced Pluripotent Stem Cells
LC-MS/MS	Liquid Chromatography Tandem Mass Spectrometry
MAP	Methamphetamine-Associated Psychosis
MPST	Mercaptopyruvate Sulfurtransferase
mRNA	Messenger RNA
PIXIE	Particle-Induced X-Ray Emission
PPAR	Peroxisome Proliferator-Activated Receptor
PUFA	Polyunsaturated Fatty Acid
qRT-PCR	Real-Time Quantitative Reverse Transcription Polymerase Chain Reaction
RIA	Radioimmunoassay
RSP	Risperidone
RXR	Retinoid X Receptor
SCID	Structured Clinical Interview for The Diagnostic and Statistical Manual of Mental Disorders
SCZ	Schizophrenia
SFI1	Centrosome-Related Gene Stress Resistance Protein 1
Δ9-THC	Delta-9 tetrahydrocannabinol
UHPLC-MS/MS	Ultra-High Performance Liquid Chromatography-Tandem Mass Spectrometry System

## References

[1] Howes O.D., Bukala B.R., Beck K. (2024). Schizophrenia: From neurochemistry to circuits, symptoms and treatments. Nat. Rev. Neurol..

[2] Jauhar S., Johnstone M., McKenna P.J. (2022). Schizophrenia. Lancet.

[3] McCutcheon R.A., Reis Marques T., Howes O.D. (2020). Schizophrenia—An Overview. JAMA Psychiatry.

[4] Jaaro-Peled H., Sawa A. (2020). Neurodevelopmental Factors in Schizophrenia. Psychiatr. Clin. N. Am..

[5] Striebel J.M. (2024). What is schizophrenia—Symptomatology. CNS Spectr..

[6] Fond G., d’Albis M.-A., Jamain S., Tamouza R., Arango C., Fleischhacker W.W., Glenthoj B., Leweke M., Lewis S., McGuire P. (2015). The Promise of Biological Markers for Treatment Response in First-Episode Psychosis: A Systematic Review. Schizophr. Bull..

[7] Chan M.K., Krebs M.-O., Cox D., Guest P.C., Yolken R.H., Rahmoune H., Rothermundt M., Steiner J., Leweke F.M., Van Beveren N.J.M. (2015). Development of a blood-based molecular biomarker test for identification of schizophrenia before disease onset. Transl. Psychiatry.

[8] Shahzade C., Chun J., DeLisi L.E., Manschreck T.C. (2018). Patterns in adolescent cannabis use predict the onset and symptom structure of schizophrenia-spectrum disorder. Schizophr. Res..

[9] Jaeger M., Briner D., Kawohl W., Seifritz E., Baumgartner-Nietlisbach G. (2015). Psychosocial functioning of individuals with schizophrenia in community housing facilities and the psychiatric hospital in Zurich. Psychiatry Res..

[10] Larsen T.K., Melle I., Auestad B., Haahr U., Joa I., Johannessen J.O., Opjordsmoen S., Rund B.R., Rossberg J.I., Simonsen E. (2011). Early detection of psychosis: Positive effects on 5-year outcome. Psychol. Med..

[11] Häfner H., Maurer K. (2006). Early detection of schizophrenia: Current evidence and future perspectives. World Psychiatry Off. J. World Psychiatr. Assoc. (WPA).

[12] Walker E.F. (2002). The Role of Endogenous and Exogenous Risk Factors in the Genesis of Schizophrenia. The Effects of Parental Dysfunction on Children.

[13] Ren S., Sun Z., Yang J. (2023). The use of biochemical indexes in hair for clinical studies of psychiatric diseases: What can we learn about mental disease from hair?. J. Psychiatr. Res..

[14] Maekawa M., Yamada K., Toyoshima M., Ohnishi T., Iwayama Y., Shimamoto C., Toyota T., Nozaki Y., Balan S., Matsuzaki H. (2015). Utility of Scalp Hair Follicles as a Novel Source of Biomarker Genes for Psychiatric Illnesses. Biol. Psychiatry.

[15] Herane Vives A., De Angel V., Papadopoulos A., Strawbridge R., Wise T., Young A.H., Arnone D., Cleare A.J. (2015). The relationship between cortisol, stress and psychiatric illness: New insights using hair analysis. J. Psychiatr. Res..

[16] Fišar Z. (2023). Biological hypotheses, risk factors, and biomarkers of schizophrenia. Prog. Neuro-Psychopharmacol. Biol. Psychiatry.

[17] Berdeville C.H.D.S.F., Silva-Amaral D., Dalgalarrondo P., Banzato C.E.M., Martins-de-Souza D. (2025). A scoping review of protein biomarkers for schizophrenia: State of progress, underlying biology, and methodological considerations. Neurosci. Biobehav. Rev..

[18] Abi-Dargham A., Moeller S.J., Ali F., DeLorenzo C., Domschke K., Horga G., Jutla A., Kotov R., Paulus M.P., Rubio J.M. (2023). Candidate biomarkers in psychiatric disorders: State of the field. World Psychiatry.

[19] Hakami M.A. (2025). Molecular signatures and emerging therapeutic targets in schizophrenia: A biomarker-centric perspective in psychiatric disorders. Schizophr. Res..

[20] Cao T., Li N., Cai H. (2020). Candidate metabolic biomarkers for schizophrenia in CNS and periphery: Do any possible associations exist?. Schizophr. Res..

[21] Rømer T.B., Jeppesen R., Christensen R.H.B., Benros M.E. (2023). Biomarkers in the cerebrospinal fluid of patients with psychotic disorders compared to healthy controls: A systematic review and meta-analysis. Mol. Psychiatry.

[22] Shkreli L., Woud M.L., Bergunde L., Schindler-Gmelch L., Blackwell S.E., Kirschbaum C., Kessler H., Steudte-Schmiedgen S. (2025). The role of long-term hair steroids as diagnostic and intervention-related biomarkers in a multimorbid inpatient sample with posttraumatic stress disorder. Eur. J. Psychotraumatol..

[23] Mohammadi A., Rashidi E., Amooeian V.G. (2018). Brain, blood, cerebrospinal fluid, and serum biomarkers in schizophrenia. Psychiatry Res..

[24] Tian Y.E., Di Biase M.A., Mosley P.E., Lupton M.K., Xia Y., Fripp J., Breakspear M., Cropley V., Zalesky A. (2023). Evaluation of Brain-Body Health in Individuals with Common Neuropsychiatric Disorders. JAMA Psychiatry.

[25] Davison J., O’Gorman A., Brennan L., Cotter D.R. (2018). A systematic review of metabolite biomarkers of schizophrenia. Schizophr. Res..

[26] Perkovic M., Erjavec G., Strac D., Uzun S., Kozumplik O., Pivac N. (2017). Theranostic Biomarkers for Schizophrenia. Int. J. Mol. Sci..

[27] Molavi N., Ghaderi A., Banafshe H. (2020). Determination of thallium in urine, blood, and hair in illicit opioid users in Iran. Hum. Exp. Toxicol..

[28] Błach-Legawiec I., Emich-Widera E., Bibrzycka A., Marszał E., Woś H. (2002). Concentrations of cadmium in blood and urine and their contents in the hair of children from Katowice Murcki. Wiadomosci Lekarskie.

[29] Wester V.L., Van Rossum E.F.C. (2015). Clinical applications of cortisol measurements in hair. Eur. J. Endocrinol..

[30] Cooper G.A.A., Kronstrand R., Kintz P. (2012). Society of Hair Testing guidelines for drug testing in hair. Forensic Sci. Int..

[31] Van Dorn R.A., Desmarais S.L., Scott Young M., Sellers B.G., Swartz M.S. (2012). Assessing illicit drug use among adults with schizophrenia. Psychiatry Res..

[32] Kogler L., Wang R., Luther T., Hofer A., Frajo-Apor B., Derntl B. (2025). Cortisol in schizophrenia spectrum disorders: A comprehensive meta-analysis. Front. Neuroendocrinol..

[33] Saghazadeh A., Mahmoudi M., Shahrokhi S., Mojarrad M., Dastmardi M., Mirbeyk M., Rezaei N. (2020). Trace elements in schizophrenia: A systematic review and meta-analysis of 39 studies (N = 5151 participants). Nutr. Rev..

[34] Bahorik A.L., Newhill C.E., Eack S.M. (2014). Neurocognitive Functioning of Individuals with Schizophrenia: Using and Not Using Drugs. Schizophr. Bull..

[35] Qi D., Wang W., Chu L., Wu Y., Wang W., Zhu M., Yuan L., Gao W., Deng H. (2024). Associations of schizophrenia with the activities of the HPA and HPG axes and their interactions characterized by hair-based biomarkers. Psychoneuroendocrinology.

[36] Murtada K., De Andrés F., Galván I., Ríos Á., Zougagh M. (2020). LC-MS determination of catecholamines and related metabolites in red deer urine and hair extracted using magnetic multi-walled carbon nanotube poly(styrene-co-divinylbenzene) composite. J. Chromatogr. B.

[37] Sun X., Wang L., Yang F., Ren J., Jiang P., Liu H., Li H., Li C., Zhang C. (2019). Correlation of hair risperidone concentration and serum level among patients with schizophrenia. Gen. Psychiatry.

[38] Maekawa M., Watanabe A., Iwayama Y., Kimura T., Hamazaki K., Balan S., Ohba H., Hisano Y., Nozaki Y., Ohnishi T. (2017). Polyunsaturated fatty acid deficiency during neurodevelopment in mice models the prodromal state of schizophrenia through epigenetic changes in nuclear receptor genes. Transl. Psychiatry.

[39] Robicsek O., Karry R., Petit I., Salman-Kesner N., Müller F.-J., Klein E., Aberdam D., Ben-Shachar D. (2013). Abnormal neuronal differentiation and mitochondrial dysfunction in hair follicle-derived induced pluripotent stem cells of schizophrenia patients. Mol. Psychiatry.

[40] Robin J., Lefeuvre S., Guihenneuc J., Cambien G., Dupuis A., Venisse N. (2024). Analytical methods and biomonitoring results in hair for the assessment of exposure to endocrine-disrupting chemicals: A literature review. Chemosphere.

[41] Zhang S., Yan X., Tang B., Luo W., Chen S., Luo X., Zheng J., Mai B., Yu Y. (2023). Human hair as a noninvasive matrix to assess exposure to micro-organic contaminants: State of the art review. Sci. Total Environ..

[42] Chen Y., Guo J., Xing S., Yu H., Huan T. (2021). Global-Scale Metabolomic Profiling of Human Hair for Simultaneous Monitoring of Endogenous Metabolome, Short- and Long-Term Exposome. Front. Chem..

[43] Musshoff F., Driever F., Lachenmeier K., Lachenmeier D.W., Banger M., Madea B. (2006). Results of hair analyses for drugs of abuse and comparison with self-reports and urine tests. Forensic Sci. Int..

[44] Binz T.M., Baumgartner M.R. (2016). Haaranalyse zum retrospektiven und prospektiven Konsum-Monitoring: Substanzmissbrauch, Abstinenz- und Compliancekontrolle. Praxis.

[45] Kempson I.M., Lombi E. (2011). Hair analysis as a biomonitor for toxicology, disease and health status. Chem. Soc. Rev..

[46] Wada Y., Maekawa M., Ohnishi T., Balan S., Matsuoka S., Iwamoto K., Iwayama Y., Ohba H., Watanabe A., Hisano Y. (2020). Peroxisome proliferator-activated receptor α as a novel therapeutic target for schizophrenia. eBioMedicine.

[47] Mlili N.E., Ahabrach H., Cauli O. (2023). Hair Cortisol Concentration as a Biomarker of Symptoms of Depressionin the Perinatal Period. CNS Neurol. Disord.—Drug Targets.

[48] Elnazer H.Y., Lau L.C.K., Amaro H., Baldwin D.S. (2021). Hair cortisol concentration in anxiety disorders: Exploration of relationships with symptom severity and inflammatory markers. Acta Neuropsychiatr..

[49] Ran R., Zhong X., Yang Y., Tang X., Shi M., Jiang X., Lin A., Gan X., Yu T., Hu L. (2023). Metabolomic profiling identifies hair as a robust biological sample for identifying women with cervical cancer. Med. Oncol..

[50] Faresjö Å., Theodorsson E., Stomby A., Quist H., Jones M.P., Östgren C.J., Dahlqvist P., Faresjö T. (2024). Higher hair cortisol levels associated with previous cardiovascular events and cardiovascular risks in a large cross-sectional population study. BMC Cardiovasc. Disord..

[51] Marrinan S., Roman-Urrestarazu A., Naughton D., Levari E., Collins J., Chilcott R., Bersani G., Corazza O. (2017). Hair analysis for the detection of drug use—Is there potential for evasion?. Hum. Psychopharmacol. Clin. Exp..

[52] Wennig R. (2000). Potential problems with the interpretation of hair analysis results. Forensic Sci. Int..

[53] Liao Q., Huang L., Yang J., Zhang S., Cai F., Tang B., Li L., Qin R., Yan X., Luo W. (2025). Retrospective prediction of environmental emerging contaminants exposure using human hair: Insights into suitability, reliability, and availability. J. Hazard. Mater..

[54] Steen N.E., Methlie P., Lorentzen S., Hope S., Barrett E.A., Larsson S., Mork E., Almås B., Løvås K., Agartz I. (2011). Increased Systemic Cortisol Metabolism in Patients with Schizophrenia and Bipolar Disorder: A Mechanism for Increased Stress Vulnerability?. J. Clin. Psychiatry.

[55] Arinami H., Watanabe Y., Suzuki Y., Tajiri M., Tsuneyama N., Someya T. (2023). Serum cortisol and insulin-like growth factor 1 levels in major depressive disorder and schizophrenia. Sci. Rep..

[56] Cao B., Yan L., Ma J., Jin M., Park C., Nozari Y., Kazmierczak O.P., Zuckerman H., Lee Y., Pan Z. (2019). Comparison of serum essential trace metals between patients with schizophrenia and healthy controls. J. Trace Elem. Med. Biol..

[57] Cohen A.N., Collins G., Nucifora F.C., Strobel R., Wait D.B., Young A.S. (2018). Clinical Consensus Recommendations for Urine Testing of Adherence to Antipsychotics Among People with Serious Mental Illness. Psychiatr. Serv..

[58] Burghardt K.J., Mando W., Seyoum B., Yi Z., Burghardt P.R. (2022). The effect of antipsychotic treatment on hormonal, inflammatory, and metabolic biomarkers in healthy volunteers: A systematic review and meta-analysis. Pharmacother. J. Hum. Pharmacol. Drug Ther..

[59] Hart X.M., Gründer G., Ansermot N., Conca A., Corruble E., Crettol S., Cumming P., Frajerman A., Hefner G., Howes O. (2024). Optimisation of pharmacotherapy in psychiatry through therapeutic drug monitoring, molecular brain imaging and pharmacogenetic tests: Focus on antipsychotics. World J. Biol. Psychiatry.

[60] Gururajan A., Manning E.E., Klug M., Van Den Buuse M. (2012). Drugs of abuse and increased risk of psychosis development. Aust. N. Z. J. Psychiatry.

[61] Casadio P., Fernandes C., Murray R.M., Di Forti M. (2011). Cannabis use in young people: The risk for schizophrenia. Neurosci. Biobehav. Rev..

[62] Tomášková A., Šlamberová R., Černá M. (2020). Influence of Prenatal Methamphetamine Abuse on the Brain. Epigenomes.

[63] Chellan P., Sadler P.J. (2015). The elements of life and medicines. Philos. Trans. R. Soc. A Math. Phys. Eng. Sci..

[64] Wang B., Fang T., Chen H. (2023). Zinc and Central Nervous System Disorders. Nutrients.

[65] Shayganfard M. (2022). Are Essential Trace Elements Effective in Modulation of Mental Disorders? Update and Perspectives. Biol. Trace Elem. Res..

[66] Borella P., Bargellini A., Caselgrandi E., Piccinini L. (1997). Observations on the Use of Plasma, Hair and Tissue to Evaluate Trace Element Status in Cancer. J. Trace Elem. Med. Biol..

[67] Schmitt A., Uhrig S., Spanagel R., Von Wilmsdorff M., Kalman J.L., Schneider-Axmann T., Falkai P., Hansson A.C. (2022). Post-mortem gene expression of calcium channels Cav1.2 and Cav1.3 in schizophrenia. Eur. Arch. Psychiatry Clin. Neurosci..

[68] Bojarski L., Debowska K., Wojda U. (2010). In vitro findings of alterations in intracellular calcium homeostasis in schizophrenia. Prog. Neuro-Psychopharmacol. Biol. Psychiatry.

[69] Zhang Z., Lin H., Feng Z., Xie H., Liu P., Shu Y., Jia Z., Zhang S. (2023). Impaired calcium channel function and pronounced hippocampal atrophy in a schizophrenia patient with cognitive impairment carrying Presenilin-2 Ser130Leu mutation: A case report and literature review. Schizophr. Res..

[70] Vidal-Domènech F., Riquelme G., Pinacho R., Rodriguez-Mias R., Vera A., Monje A., Ferrer I., Callado L.F., Meana J.J., Villén J. (2020). Calcium-binding proteins are altered in the cerebellum in schizophrenia. PLoS ONE.

[71] Kang Y., Sheng L., Li J. (2025). HINT1 promotes neuronal apoptosis and triggers schizophrenia-like behavior in rats. Behav. Brain Res..

[72] Jarskog L.F., Selinger E.S., Lieberman J.A., Gilmore J.H. (2004). Apoptotic Proteins in the Temporal Cortex in Schizophrenia: High Bax/Bcl-2 Ratio Without Caspase-3 Activation. Am. J. Psychiatry.

[73] Parellada E., Gassó P. (2021). Glutamate and microglia activation as a driver of dendritic apoptosis: A core pathophysiological mechanism to understand schizophrenia. Transl. Psychiatry.

[74] Sonnenschein S.F., Parr A.C., Larsen B., Calabro F.J., Foran W., Eack S.M., Luna B., Sarpal D.K. (2022). Subcortical brain iron deposition in individuals with schizophrenia. J. Psychiatr. Res..

[75] Lotan A., Luza S., Opazo C.M., Ayton S., Lane D.J.R., Mancuso S., Pereira A., Sundram S., Weickert C.S., Bousman C. (2023). Perturbed iron biology in the prefrontal cortex of people with schizophrenia. Mol. Psychiatry.

[76] Maxwell A.M., Rao R.B. (2022). Perinatal iron deficiency as an early risk factor for schizophrenia. Nutr. Neurosci..

[77] Tabata K., Miyashita M., Yamasaki S., Toriumi K., Ando S., Suzuki K., Endo K., Morimoto Y., Tomita Y., Yamaguchi S. (2022). Hair zinc levels and psychosis risk among adolescents. Schizophrenia.

[78] Rahman M.A., Azad M.A.K., Hossain M.I., Qusar M.M.A.S., Bari W., Begum F., Huq S.M.I., Hasnat A. (2009). Zinc, Manganese, Calcium, Copper, and Cadmium Level in Scalp Hair Samples of Schizophrenic Patients. Biol. Trace Elem. Res..

[79] Pradeep A.S., Abdul Sattar S., Seetharami Reddy B., Durga Prasada Rao A. (2023). Levels of a select group of trace elements in scalp hair of schizophrenics by PIXE. Nucl. Instrum. Methods Phys. Res. Sect. B Beam Interact. Mater. Atoms..

[80] Starzer M.S.K., Nordentoft M., Hjorthøj C. (2018). Rates and Predictors of Conversion to Schizophrenia or Bipolar Disorder Following Substance-Induced Psychosis. Am. J. Psychiatry.

[81] Werner F.-M., Covenas R. (2018). Long-term Administration of Antipsychotic Drugs in Schizophrenia and Influence of Substance and Drug Abuse on the Disease Outcome. Curr. Drug Abus. Rev..

[82] Olfson M., Gerhard T., Huang C., Crystal S., Stroup T.S. (2015). Premature Mortality Among Adults with Schizophrenia in the United States. JAMA Psychiatry.

[83] Schiffer B., Müller B.W., Scherbaum N., Forsting M., Wiltfang J., Leygraf N., Gizewski E.R. (2010). Impulsivity-related brain volume deficits in schizophrenia-addiction comorbidity. Brain.

[84] Smith J., Hucker S. (1994). Schizophrenia and Substance Abuse. Br. J. Psychiatry.

[85] Bahorik A.L., Newhill C.E., Queen C.C., Eack S.M. (2014). Under-reporting of drug use among individuals with schizophrenia: Prevalence and predictors. Psychol. Med..

[86] Stone A.M., Greenstein R.A., Gamble G., McLellan A.T. (1993). Cocaine Use by Schizophrenic Outpatients Who Receive Depot Neuroleptic Medication. Psychiatr. Serv..

[87] Møller T., Linaker O.M. (2010). Using brief self-reports and clinician scales to screen for substance use disorders in psychotic patients. Nord. J. Psychiatry.

[88] McPhillips M.A., Kelly F.J., Barnes T.R.E., Duke P.J., Gene-Cos N., Clark K. (1997). Detecting comorbid substance misuse among people with schizophrenia in the community: A study comparing the results of questionnaires with analysis of hair and urine. Schizophr. Res..

[89] Swartz M.S., Swanson J.W., Hannon M.J. (2003). Detection of Illicit Substance Use Among Persons with Schizophrenia by Radioimmunoassay of Hair. Psychiatr. Serv..

[90] Nestoros J.N., Vakonaki E., Tzatzarakis M.N., Alegakis A., Skondras M.D., Tsatsakis A.M. (2017). Long lasting effects of chronic heavy cannabis abuse. Am. J. Addict..

[91] Morgan C.J.A., Curran H.V. (2008). Effects of cannabidiol on schizophrenia-like symptoms in people who use cannabis. Br. J. Psychiatry.

[92] Lamyai W., Pono K., Indrakamhaeng D., Saengsin A., Songhong N., Khuwuthyakorn P., Sribanditmongkol P., Junkuy A., Srisurapanont M. (2019). Risks of psychosis in methamphetamine users: Cross-sectional study in Thailand. BMJ Open.

[93] Garcia-Bournissen F., Rokach B., Karaskov T., Koren G. (2007). Methamphetamine detection in maternal and neonatal hair: Implications for fetal safety. Arch. Dis. Child.—Fetal Neonatal Ed..

[94] Meyer J.M. (2025). How antipsychotics work in schizophrenia: A primer on mechanisms. CNS Spectr..

[95] Howes O.D., Dawkins E., Lobo M.C., Kaar S.J., Beck K. (2024). New Drug Treatments for Schizophrenia: A Review of Approaches to Target Circuit Dysfunction. Biol. Psychiatry.

[96] Curto M., Fazio F., Ulivieri M., Navari S., Lionetto L., Baldessarini R.J. (2021). Improving adherence to pharmacological treatment for schizophrenia: A systematic assessment. Expert Opin. Pharmacother..

[97] Tiihonen J., Tanskanen A., Taipale H. (2018). 20-Year Nationwide Follow-Up Study on Discontinuation of Antipsychotic Treatment in First-Episode Schizophrenia. Am. J. Psychiatry.

[98] Leucht S., Priller J., Davis J.M. (2024). Antipsychotic Drugs: A Concise Review of History, Classification, Indications, Mechanism, Efficacy, Side Effects, Dosing, and Clinical Application. Am. J. Psychiatry.

[99] Kronstrand R., Roman M., Hedman M., Ahlner J., Dizdar N. (2007). Dose–hair concentration relationship and pigmentation effects in patients on low-dose clozapine. Forensic Sci. Med. Pathol..

[100] Kintz P., Villain M., Cirimele V. (2006). Hair Analysis for Drug Detection. Ther. Drug Monit..

[101] Schoretsanitis G., Kane J.M., Correll C.U., Marder S.R., Citrome L., Newcomer J.W., Robinson D.G., Goff D.C., Kelly D.L., Freudenreich O. (2020). Blood Levels to Optimize Antipsychotic Treatment in Clinical Practice: A Joint Consensus Statement of the American Society of Clinical Psychopharmacology and the Therapeutic Drug Monitoring Task Force of the Arbeitsgemeinschaft für Neuropsychopharmakologie und Pharmakopsychiatrie. J. Clin. Psychiatry.

[102] Cirimele V., Kintz P., Gosselin O., Ludes B. (2000). Clozapine dose–concentration relationships in plasma, hair and sweat specimens of schizophrenic patients. Forensic Sci. Int..

[103] Wang X., Zhuo Y., Tang X., Qiang H., Liu W., Wu H., Xiang P., Duan G., Shen M. (2019). Segmental analysis of antidepressant and antipsychotic drugs in the hair of schizophrenic patients. Drug Test. Anal..

[104] Cobo-Golpe M., de-Castro-Ríos A., Cruz A., Páramo M., López-Rivadulla M., Lendoiro E. (2020). Determination of antipsychotic drugs in nails and hair by liquid chromatography tandem mass spectrometry and evaluation of their incorporation into keratinized matrices. J. Pharm. Biomed. Anal..

[105] Yang H., Liu C., Zhu C., Zheng Y., Li J., Zhu Q., Wang H., Fang X., Liu Q., Liang M. (2023). Determination of ten antipsychotics in blood, hair and nails: Validation of a LC–MS/MS method and forensic application of keratinized matrix analysis. J. Pharm. Biomed. Anal..

[106] Schneider S., Sibille E., Yegles M., Neels H., Wennig R., Mühe A. (2009). Time resolved analysis of risperidone and 9-hydroxy-risperidone in hair using LC/MS-MS. J. Chromatogr. B.

[107] Ramírez Fernández M.D.M., Baumgartner W.A., Wille S.M.R., Farabee D., Samyn N., Baumgartner A.M. (2020). A different insight in hair analysis: Simultaneous measurement of antipsychotic drugs and metabolites in the protein and melanin fraction of hair from criminal justice patients. Forensic Sci. Int..

[108] Balasamy S., Atchudan R., Arya S., Gunasekaran B.M., Nesakumar N., Sundramoorthy A.K. (2024). Cortisol: Biosensing and detection strategies. Clin. Chim. Acta.

[109] Aas M., Pizzagalli D.A., Laskemoen J.F., Reponen E.J., Ueland T., Melle I., Agartz I., Steen N.E., Andreassen O.A. (2019). Elevated hair cortisol is associated with childhood maltreatment and cognitive impairment in schizophrenia and in bipolar disorders. Schizophr. Res..

[110] Altamura A.C., Boin F., Maes M. (1999). HPA axis and cytokines dysregulation in schizophrenia: Potential implications for the antipsychotic treatment. Eur. Neuropsychopharmacol..

[111] Zhu X., Zhu Y., Huang J., Zhou Y., Tong J., Zhang P., Luo X., Chen S., Tian B., Tan S. (2022). Abnormal cortisol profile during psychosocial stress among patients with schizophrenia in a Chinese population. Sci. Rep..

[112] Stalder T., Steudte-Schmiedgen S., Alexander N., Klucken T., Vater A., Wichmann S., Kirschbaum C., Miller R. (2017). Stress-related and basic determinants of hair cortisol in humans: A meta-analysis. Psychoneuroendocrinology.

[113] Girshkin L., Matheson S.L., Shepherd A.M., Green M.J. (2014). Morning cortisol levels in schizophrenia and bipolar disorder: A meta-analysis. Psychoneuroendocrinology.

[114] Koumantarou Malisiova E., Mourikis I., Darviri C., Nicolaides N.C., Zervas I.M., Papageorgiou C., Chrousos G.P. (2021). Hair cortisol concentrations in mental disorders: A systematic review. Physiol. Behav..

[115] Yang F., Hong X., Tao J., Chen Y., Zhang Y., Xiao H. (2021). Hair cortisol, social support, personality traits, and clinical course: Differences in schizophrenia and bipolar disorder. Brain Behav..

[116] Yang F., Cao X., Sun X., Wen H., Qiu J., Xiao H. (2020). Hair Cortisol Is Associated with Social Support and Symptoms in Schizophrenia. Front. Psychiatry.

[117] Hirt V., Schalinski I., Rockstroh B. (2019). Decoding the impact of adverse childhood experiences on the progression of schizophrenia. Ment. Health Prev..

[118] Van Den Heuvel L.L., Smit A.M., Stalder T., Kirschbaum C., Seedat S., Emsley R. (2022). Hair cortisol levels in schizophrenia and metabolic syndrome. Early Interv. Psychiatry.

[119] Nyström-Hansen M., Andersen M.S., Davidsen K.A., Roehder K., Trier C., Nayberg E., Lyons-Ruth K., Harder S. (2024). Hair cortisol concentrations in pregnant women with bipolar, depressive, or schizophrenic spectrum disorders. Arch. Women’s Ment. Health.

[120] Andrade E.H., Rizzo L.B., Noto C., Ota V.K., Gadelha A., Daruy-Filho L., Tasso B.D.C., Mansur R.B., Cordeiro Q., Belangero S.I. (2016). Hair cortisol in drug-naïve first-episode individuals with psychosis. Rev. Bras. Psiquiatr..

[121] Söder E., Clamor A., Lincoln T.M. (2019). Hair cortisol concentrations as an indicator of potential HPA axis hyperactivation in risk for psychosis. Schizophr. Res..

[122] Rietschel L., Streit F., Zhu G., McAloney K., Kirschbaum C., Frank J., Hansell N.K., Wright M.J., McGrath J.J., Witt S.H. (2016). Hair Cortisol and Its Association with Psychological Risk Factors for Psychiatric Disorders: A Pilot Study in Adolescent Twins. Twin Res. Hum. Genet..

[123] Streit F., Memic A., Hasandedić L., Rietschel L., Frank J., Lang M., Witt S.H., Forstner A.J., Degenhardt F., Wüst S. (2016). Perceived stress and hair cortisol: Differences in bipolar disorder and schizophrenia. Psychoneuroendocrinology.

[124] Brandt J.M., Hemager N., Ellersgaard D., Gregersen M., Søndergaard A., Ohland J., Søborg Spang K., Christiani C., Burton B.K., Greve A. (2023). Hair cortisol concentrations and daily life stress in 7-year-old children at familial high-risk of schizophrenia or bipolar disorder. The Danish High Risk and Resilience Study—VIA 7. Prog. Neuro-Psychopharmacol. Biol. Psychiatry.

[125] Mikulska J., Juszczyk G., Gawrońska-Grzywacz M., Herbet M. (2021). HPA Axis in the Pathomechanism of Depression and Schizophrenia: New Therapeutic Strategies Based on Its Participation. Brain Sci..

[126] Stahl S.M. (2018). Beyond the dopamine hypothesis of schizophrenia to three neural networks of psychosis: Dopamine, serotonin, and glutamate. CNS Spectr..

[127] Wijtenburg S.A., Yang S., Fischer B.A., Rowland L.M. (2015). In vivo assessment of neurotransmitters and modulators with magnetic resonance spectroscopy: Application to schizophrenia. Neurosci. Biobehav. Rev..

[128] Kumar D., Sinha S.N., Gouda B. (2024). Novel LC-MS/MS Method for Simultaneous Determination of Monoamine Neurotransmitters and Metabolites in Human Samples. J. Am. Soc. Mass Spectrom..

[129] Gao L., Yuan H., Zhu J., Hara K., Liu J. (2016). Determination of Tyramine in Hair Samples by GC–MS. Chromatographia.

[130] Cuevas-Diaz Duran R., Martinez-Ledesma E., Garcia-Garcia M., Bajo Gauzin D., Sarro-Ramírez A., Gonzalez-Carrillo C., Rodríguez-Sardin D., Fuentes A., Cardenas-Lopez A. (2024). The Biology and Genomics of Human Hair Follicles: A Focus on Androgenetic Alopecia. Int. J. Mol. Sci..

[131] Ide M., Ohnishi T., Toyoshima M., Balan S., Maekawa M., Shimamoto-Mitsuyama C., Iwayama Y., Ohba H., Watanabe A., Ishii T. (2019). Excess hydrogen sulfide and polysulfides production underlies a schizophrenia pathophysiology. EMBO Mol. Med..

[132] Matsuura A., Ishima T., Fujita Y., Iwayama Y., Hasegawa S., Kawahara-Miki R., Maekawa M., Toyoshima M., Ushida Y., Suganuma H. (2018). Dietary glucoraphanin prevents the onset of psychosis in the adult offspring after maternal immune activation. Sci. Rep..

[133] Faludi G., Mirnics K. (2011). Synaptic changes in the brain of subjects with schizophrenia. Int. J. Dev. Neurosci..

[134] Weickert C.S., Weickert T.W., Pillai A., Buckley P.F. (2013). Biomarkers in Schizophrenia: A Brief Conceptual Consideration. Dis. Markers.

[135] Stalder T., Kirschbaum C. (2012). Analysis of cortisol in hair—State of the art and future directions. Brain Behav. Immun..

[136] Bade A., Yadav P., Zhang L., Naidu Bypaneni R., Xu M., Glass T.E. (2024). Imaging Neurotransmitters with Small-Molecule Fluorescent Probes. Angew. Chem. Int. Ed..

[137] Lovinger D.M. (2008). Communication networks in the brain: Neurons, receptors, neurotransmitters, and alcohol. Alcohol Res. Health J. Natl. Inst. Alcohol Abus. Alcohol..

[138] Brennenstuhl H., Jung-Klawitter S., Assmann B., Opladen T. (2019). Inherited Disorders of Neurotransmitters: Classification and Practical Approaches for Diagnosis and Treatment. Neuropediatrics.

[139] Shimamoto C., Ohnishi T., Maekawa M., Watanabe A., Ohba H., Arai R., Iwayama Y., Hisano Y., Toyota T., Toyoshima M. (2014). Functional characterization of FABP3, 5 and 7 gene variants identified in schizophrenia and autism spectrum disorder and mouse behavioral studies. Hum. Mol. Genet..

[140] Vincenti M., Kintz P. (2015). New Challenges and Perspectives in Hair Analysis. Hair Analysis in Clinical and Forensic Toxicology.

[141] Eisenbeiss L., Binz T.M., Baumgartner M.R., Kraemer T., Steuer A.E. (2020). Cheating on forensic hair testing? Detection of potential biomarkers for cosmetically altered hair samples using untargeted hair metabolomics. Analyst.

[142] Rashaid A.B., Khasawneh Z., Alqhazo M., Nusair S., El-Khateeb M., Bashtawi B. (2021). Underivatized Amino Acid Analyses Using Liquid Chromatography-Tandem Mass Spectrometry in Scalp Hair of Children with Autism Spectrum Disorder. Int. J. Chem. Mol. Eng..

[143] Schoonover K.E., Roberts R.C. (2021). Markers of copper transport in the cingulum bundle in schizophrenia. Schizophr. Res..

[144] Schoonover K.E., Queern S.L., Lapi S.E., Roberts R.C. (2020). Impaired copper transport in schizophrenia results in a copper-deficient brain state: A new side to the dysbindin story. World J. Biol. Psychiatry.

[145] Konradi C. (2011). Myelin copper and the cuprizone model of schizophrenia. Front. Biosci..

[146] Tyrer S.P., Delves H.T., Weller M.P. (1979). CSF copper in schizophrenia. Am. J. Psychiatry.

[147] Yanik M., Kocyigit A., Tutkun H., Vural H., Herken H. (2004). Plasma Manganese, Selenium, Zinc, Copper, and Iron Concentrations in Patients with Schizophrenia. Biol. Trace Elem. Res..

[148] Gillin J.C., Carpenter W.T., Hambidge K.M., Wyatt R.J., Henkin R.I. (1982). Zinc and copper in patients with schizophrenia. L’Encephale.

[149] Joe P., Petrilli M., Malaspina D., Weissman J. (2018). Zinc in schizophrenia: A meta-analysis. Gen. Hosp. Psychiatry.

[150] Petrilli M.A., Kranz T.M., Kleinhaus K., Joe P., Getz M., Johnson P., Chao M.V., Malaspina D. (2017). The Emerging Role for Zinc in Depression and Psychosis. Front. Pharmacol..

[151] Baj J., Forma A., Sitarz E., Karakuła K., Flieger W., Sitarz M., Grochowski C., Maciejewski R., Karakula-Juchnowicz H. (2020). Beyond the Mind—Serum Trace Element Levels in Schizophrenic Patients: A Systematic Review. Int. J. Mol. Sci..

[152] Potkin S.G., Shore D., Torrey E.F., Weinberger D.R., Gillin J.C., Henkin R.I., Agarwal R.P., Wyatt R.J. (1982). Cerebrospinal fluid zinc concentrations in ex-heroin addicts and patients with schizophrenia: Some preliminary observations. Biol. Psychiatry.

[153] Lehmann H.E. (1951). Studies on the iron content of cerebrospinal fluid in different psychotic conditions. Arch. Neurol. Psychiatry.

[154] Yassa R., Nair N.P.V., Schwartz G. (1979). Plasma Magnesium in Chronic Schizophrenia. Int. Pharmacopsychiatry.

[155] Wiser M., Levkowitch Y., Neuman M., Yehuda S. (1994). Decrease of Serum Iron in Acutely Psychotic Schizophrenic Patients. Int. J. Neurosci..

[156] Kim S.-W., Stewart R., Park W.-Y., Jhon M., Lee J.-Y., Kim S.-Y., Kim J.-M., Amminger P., Chung Y.-C., Yoon J.-S. (2018). Latent Iron Deficiency as a Marker of Negative Symptoms in Patients with First-Episode Schizophrenia Spectrum Disorder. Nutrients.

[157] Berridge M.J. (2013). Dysregulation of neural calcium signaling in Alzheimer disease, bipolar disorder and schizophrenia. Prion.

[158] Berridge M.J. (2014). Calcium signalling and psychiatric disease: Bipolar disorder and schizophrenia. Cell Tissue Res..

[159] Jimerson D.C., Post R.M., Carman J.S., van Kammen D.P., Wood J.H., Goodwin F.K., Bunney W.E. (1979). CSF calcium: Clinical correlates in affective illness and schizophrenia. Biol. Psychiatry.

[160] Nechifor M., Vaideanu C., Palamaru I., Borza C., Mindreci I. (2004). The Influence of Some Antipsychotics on Erythrocyte Magnesium and Plasma Magnesium, Calcium, Copper and Zinc in Patients with Paranoid Schizophrenia. J. Am. Coll. Nutr..

[161] Wyszogrodzka-Kucharska A., Rabe-Jabłońska J. (2005). Calcium balance and regulation in schizophrenic patients treated with second generation antipsychotics. Psychiatr. Pol..

[162] Orisakwe O. (2014). The role of lead and cadmium in psychiatry. N. Am. J. Med. Sci..

[163] Shen B., Lu R., Lv M., Chen J., Li J., Long J., Cai H., Su L., Gong Z. (2024). Association between the levels of toxic heavy metals and schizophrenia in the population of Guangxi, China: A case-control study. Environ. Pollut..

[164] Arinola G., Idonije B., Akinlade K., Ihenyen O. (2010). Essential trace metals and heavy metals in newly diagnosed schizophrenic patients and those on anti-psychotic medication. J. Res. Med. Sci. Off. J. Isfahan Univ. Med. Sci..

[165] Michel T.M., Thome J., Martin D., Nara K., Zwerina S., Tatschner T., Weijers H.G., Koutsilieri E. (2004). Cu, Zn- and Mn-superoxide dismutase levels in brains of patients with schizophrenic psychosis. J. Neural Transm..

[166] Carl C. (1983). Pfeiffer; Scott LaMola Zinc and manganese in the schizophrenias. J. Orthomol. Med..

[167] Liu T., Lu Q.-B., Yan L., Guo J., Feng F., Qiu J., Wang J. (2015). Comparative Study on Serum Levels of 10 Trace Elements in Schizophrenia. PLoS ONE.

[168] Yanik M., Vural H., Kocyigit A., Tutkun H., Zoroglu S.S., Herken H., Savaş H.A., Köylü A., Akyol Ö. (2003). Is the Arginine-Nitric Oxide Pathway Involved in the Pathogenesis of Schizophrenia?. Neuropsychobiology.

[169] Kaya B., Akdağ N., Fadıllıoğlu E., Taycan S.E., Emre M.H., Unal S., Sayal A., Erdoğan H., Polat R. (2012). Şizofreni hastalarında kanda glukoz-6-fosfat dehidrogenaz aktivitesi ve element düzeyleri/Elements levels and glucose-6-phosphate dehydrogenase activity in blood of patients with schizophrenia. Dusunen Adam J. Psychiatry Neurol. Sci..

[170] Kanabrocki E.L., Case L.F., Fields T., Graham L., Miller E.B., Oester Y.T., Kaplan E. (1965). Manganese and copper levels in human urine. J. Nucl. Med. Off. Publ. Soc. Nucl. Med..

[171] Muguruza C., Lehtonen M., Aaltonen N., Morentin B., Meana J.J., Callado L.F. (2013). Quantification of endocannabinoids in postmortem brain of schizophrenic subjects. Schizophr. Res..

[172] Thoma P., Daum I. (2013). Comorbid substance use disorder in schizophrenia: A selective overview of neurobiological and cognitive underpinnings. Psychiatry Clin. Neurosci..

[173] Thornton S.L., Lo J., Clark R.F., Wu A.H.B., Gerona R.R. (2012). Simultaneous detection of multiple designer drugs in serum, urine, and CSF in a patient with prolonged psychosis. Clin. Toxicol..

[174] Brunette M.F., Roth R.M., Trask C., Khokhar J.Y., Ford J.C., Park S.H., Hickey S.M., Zeffiro T., Xie H. (2025). Randomized Laboratory Study of Single-Dose Cannabis, Dronabinol, and Placebo in Patients with Schizophrenia and Cannabis Use Disorder. Schizophr. Bull..

[175] Large M.M., Smith G., Sara G., Paton M.B., Kedzior K.K., Nielssen O.B. (2012). Meta-analysis of self-reported substance use compared with laboratory substance assay in general adult mental health settings. Int. J. Methods Psychiatr. Res..

[176] Fowler I.L., Carr V.J., Carter N.T., Lewin T.J. (1998). Patterns of Current and Lifetime Substance Use in Schizophrenia. Schizophr. Bull..

[177] Sampedro M.C., Unceta N., Gómez-Caballero A., Callado L.F., Morentin B., Goicolea M.A., Meana J.J., Barrio R.J. (2012). Screening and quantification of antipsychotic drugs in human brain tissue by liquid chromatography–tandem mass spectrometry: Application to postmortem diagnostics of forensic interest. Forensic Sci. Int..

[178] Saar E., Beyer J., Gerostamoulos D., Drummer O.H. (2012). The analysis of antipsychotic drugs in human matrices using LC-MS(/MS). Drug Test. Anal..

[179] Patteet L., Cappelle D., Maudens K.E., Crunelle C.L., Sabbe B., Neels H. (2015). Advances in detection of antipsychotics in biological matrices. Clin. Chim. Acta.

[180] Skogh E., Sjödin I., Josefsson M., Dahl M.-L. (2011). High Correlation Between Serum and Cerebrospinal Fluid Olanzapine Concentrations in Patients with Schizophrenia or Schizoaffective Disorder Medicating With Oral Olanzapine as the Only Antipsychotic Drug. J. Clin. Psychopharmacol..

[181] Dziurkowska E., Wesolowski M. (2021). Cortisol as a Biomarker of Mental Disorder Severity. J. Clin. Med..

[182] Issa G., Wilson C., Terry A.V., Pillai A. (2010). An inverse relationship between cortisol and BDNF levels in schizophrenia: Data from human postmortem and animal studies. Neurobiol. Dis..

[183] Pearson A., De Vries A., Middleton S.D., Gillies F., White T.O., Armstrong I.R., Andrew R., Seckl J.R., MacLullich A.M. (2010). Cerebrospinal fluid cortisol levels are higher in patients with delirium versus controls. BMC Res. Notes.

[184] Hubbard D.B., Miller B.J. (2019). Meta-analysis of blood cortisol levels in individuals with first-episode psychosis. Psychoneuroendocrinology.

[185] Steen N.E., Methlie P., Lorentzen S., Dieset I., Aas M., Nerhus M., Haram M., Agartz I., Melle I., Berg J.P. (2014). Altered systemic cortisol metabolism in bipolar disorder and schizophrenia spectrum disorders. J. Psychiatr. Res..

[186] Steen N.E., Tesli M., Kähler A.K., Methlie P., Hope S., Barrett E.A., Larsson S., Mork E., Løvås K., Røssberg J.I. (2010). SRD5A2 is associated with increased cortisol metabolism in schizophrenia spectrum disorders. Prog. Neuro-Psychopharmacol. Biol. Psychiatry.

[187] Mackay A.V.P. (1982). Increased Brain Dopamine and Dopamine Receptors in Schizophrenia. Arch. Gen. Psychiatry.

[188] Bird E.D., Spokes E.G.S., Iversen L.L. (1979). Increased dopamine concentration in limbic areas of brain from patients dying with schizophrenia. Brain.

[189] Davis K.L., Kahn R.S., Ko G., Davidson M. (1991). Dopamine in schizophrenia: A review and reconceptualization. Am. J. Psychiatry.

[190] Moncrieff J. (2009). A Critique of the Dopamine Hypothesis of Schizophrenia and Psychosis. Harv. Rev. Psychiatry.

[191] Orhan F., Goiny M., Becklén M., Mathé L., Piehl F., Schwieler L., Fatouros-Bergman H., Farde L., Cervenka S., Sellgren C.M. (2023). CSF dopamine is elevated in first-episode psychosis and associates to symptom severity and cognitive performance. Schizophr. Res..

[192] Shiga T., Horikoshi S., Kanno K., Kanno-Nozaki K., Hikita M., Itagaki S., Miura I., Yabe H. (2020). Plasma levels of dopamine metabolite correlate with mismatch negativity in patients with schizophrenia. Psychiatry Clin. Neurosci..

[193] Van Kammen D.P., Kelley M. (1991). Dopamine and norepinephrine activity in schizophrenia. Schizophr. Res..

[194] Crowley T.J. (1978). Dopamine Excretion and Vulnerability to Drug-Induced Parkinsonism: Schizophrenic Patients. Arch. Gen. Psychiatry.

[195] Vingerhoets C., Bloemen O.J.N., Boot E., Bakker G., De Koning M.B., Da Silva Alves F., Booij J., Van Amelsvoort T.A.M.J. (2018). Dopamine in high-risk populations: A comparison of subjects with 22q11.2 deletion syndrome and subjects at ultra high-risk for psychosis. Psychiatry Res. Neuroimaging.

[196] Bleich A., Brown S.-L., Kahn R., Van Praag H.M. (1988). The Role of Serotonin in Schizophrenia. Schizophr. Bull..

[197] Quednow B.B., Geyer M.A., Halberstadt A.L. (2020). Serotonin and schizophrenia. Handbook of Behavioral Neuroscience.

[198] Kunz M., Sikora J., Krakowski M., Convit A., Cooper T.B., Volavka J. (1995). Serotonin in violent patients with schizophrenia. Psychiatry Res..

[199] Shang P., Ho A.M.-C., Tufvesson-Alm M., Lindberg D.R., Grant C.W., Orhan F., Eren F., Bhat M., Engberg G., Schwieler L. (2022). Identification of cerebrospinal fluid and serum metabolomic biomarkers in first episode psychosis patients. Transl. Psychiatry.

[200] DeLisi L.E. (1981). Increased Whole Blood Serotonin Concentrations in Chronic Schizophrenic Patients. Arch. Gen. Psychiatry.

[201] Garelis E., Gillin J.C., Wyatt R.J., Neff N. (1975). Elevated blood serotonin concentrations in unmediated chronic schizophrenic patients: A preliminary study. Am. J. Psychiatry.

[202] Emanuele E., Colombo R., Martinelli V., Brondino N., Marini M., Boso M., Barale F., Politi P. (2010). Elevated urine levels of bufotenine in patients with autistic spectrum disorders and schizophrenia. Neuro Endocrinol. Lett..

[203] Hu W., MacDonald M.L., Elswick D.E., Sweet R.A. (2015). The glutamate hypothesis of schizophrenia: Evidence from human brain tissue studies. Ann. N. Y. Acad. Sci..

[204] Uno Y., Coyle J.T. (2019). Glutamate hypothesis in schizophrenia. Psychiatry Clin. Neurosci..

[205] Howes O., McCutcheon R., Stone J. (2015). Glutamate and dopamine in schizophrenia: An update for the 21st century. J. Psychopharmacol..

[206] Madeira C., Alheira F.V., Calcia M.A., Silva T.C.S., Tannos F.M., Vargas-Lopes C., Fisher M., Goldenstein N., Brasil M.A., Vinogradov S. (2018). Blood Levels of Glutamate and Glutamine in Recent Onset and Chronic Schizophrenia. Front. Psychiatry.

[207] Nagai T., Kirihara K., Tada M., Koshiyama D., Koike S., Suga M., Araki T., Hashimoto K., Kasai K. (2017). Reduced Mismatch Negativity is Associated with Increased Plasma Level of Glutamate in First-episode Psychosis. Sci. Rep..

[208] Song J., Viggiano A., Monda M., De Luca V. (2014). Peripheral Glutamate Levels in Schizophrenia: Evidence from a Meta-Analysis. Neuropsychobiology.

[209] Wiesel F.-A., Andersson J.L.R., Westerberg G., Wieselgren I.-M., Bjerkenstedt L., Hagenfeldt L., Långström B. (1999). Tyrosine transport is regulated differently in patients with schizophrenia. Schizophr. Res..

[210] Zaki J.K., Tomasik J., McCune J., Scherman O.A., Bahn S. (2023). Discovery of Urinary Metabolite Biomarkers of Psychiatric Disorders Using Two-Sample Mendelian Randomization. medRxiv.

[211] Pinacho R., Villalmanzo N., Meana J.J., Ferrer I., Berengueras A., Haro J.M., Villén J., Ramos B. (2016). Altered CSNK1E, FABP4 and NEFH protein levels in the dorsolateral prefrontal cortex in schizophrenia. Schizophr. Res..

[212] Martínez-Gras I., Pérez-Nievas B.G., García-Bueno B., Madrigal J.L.M., Andrés-Esteban E., Rodríguez-Jiménez R., Hoenicka J., Palomo T., Rubio G., Leza J.C. (2011). The anti-inflammatory prostaglandin 15d-PGJ2 and its nuclear receptor PPARgamma are decreased in schizophrenia. Schizophr. Res..

